# Exploring EMDR: an innovative approach with Posner Paradigm to reprocessing negative memories in a non-clinical sample

**DOI:** 10.3389/fpsyt.2025.1605608

**Published:** 2025-11-24

**Authors:** Laura Piccardi, Samuele Russo, Stefano Lasaponara, Maddalena Boccia, Chiara Riso, Emanuela Tizzani, Jessica Burrai, Anna Maria Giannini, Paola Guariglia

**Affiliations:** 1 Department of Psychology, Sapienza University of Rome, Rome, Italy; 2 IRCCS San Raffaele Cassino, Cassino, Italy; 3 Cognitive and Motor Rehabilitation and Neuroimaging Unit, IRCCS Fondazione Santa Lucia, Rome, Italy; 4 Mental Health Department, ASL Roma1, Rome, Italy; 5 Ministry of the Interior, Department of Public Security, Italian State Police, Rome, Italy; 6 Faculty of Social and Communication Sciences, Mercatorum University, Rome, Italy

**Keywords:** PTSD, traumatic memory, EMDR, attention, Posner task, psychotherapy, negative memories

## Abstract

**Introduction:**

Eye Movement Desensitization and Reprocessing (EMDR) is a structured psychotherapy primarily focused on treating individuals who have experienced distressing, traumatic events and other mental disorders. While traditionally associated with bilateral eye movements, the underlying mechanisms of EMDR remain a topic of interest. Our goal was to explore whether an endogenous attention task, specifically the Posner paradigm, which involves shifting spatial attention without eye movements, could be as effective as the conventional eye movements in processing distress memories of moderate to high intensity and provide insights into the underlying mechanisms of the technique.

**Methods:**

To achieve this, we conducted a randomized controlled trial involving 50 healthy participants, who were divided into two groups (EMDR and other engaging in Posner paradigm). Participants were tasked with recalling distress memories while undergoing their respective interventions. We measured the overall effects of both approaches on subjective units of distress (SUDs), the Impact of Event Scale-Revised (IES-R), and the Posttraumatic Stress Disorder Checklist for DSM-5 (PCL-5). Pre- and post-intervention assessments were conducted to evaluate changes in these measures.

**Results:**

Our results indicated that both the EMDR and Posner groups experienced significant reductions in scores on the SUDs, IES-R, and PCL-5, demonstrating equal effectiveness in alleviating distress associated with distress memories. Notably, the results suggest that the mechanism of attention shifting, rather than the specific modality of eye movements, plays a critical role in the therapeutic process.

**Conclusion:**

These data suggest that endogenous visuospatial tasks, such as those employed in the Posner paradigm, may serve as viable alternatives to traditional eye movements in EMDR therapy. Furthermore, our findings indicate that the simultaneous presentation of stimuli may not be a crucial aspect of EMDR’s effectiveness. This study contributes to the understanding of EMDR by highlighting the importance of attentional processes in memory processing and opens avenues for further research into alternative therapeutic techniques that leverage cognitive mechanisms. However, as this study employed a non-clinical sample of healthy participants with distress memories, caution is warranted when generalizing these findings to clinical populations with diagnosed trauma-related disorders. The implications of these findings are discussed within the broader theoretical frameworks of EMDR and attentional involvement, emphasizing the potential for integrating cognitive tasks into trauma-focused therapies.

## 1 Introduction

Posttraumatic stress disorder (PTSD) is a mental health condition that develops following exposure to a traumatic or highly stressful event. PTSD affects approximately 6% of the general population but can be present in 25–35% of individuals who have undergone significant trauma (1). Traumatic events can stem from a variety of sources, such as natural disasters, accidents, terrorist attacks, combat or war experiences, sexual assault or rape, historical trauma, domestic violence, and bullying. The DSM-5R (2) emphasizes the behavioral symptoms of PTSD and introduces four distinct diagnostic clusters instead of three. These clusters include re-experiencing, avoidance, negative cognitions and mood, and arousal. According to the DSM-5R, individuals must exhibit noticeable difficulties at work and in social situations for over a month. Re-experience, or intrusive memories, are recognized as a key symptom of post-traumatic stress disorder (2) but also occur in other conditions such as agoraphobia, social anxiety disorder, depression, bulimia nervosa, and psychosis (3); these memories often manifest as vivid visual images (4).

Most international clinical practice guidelines for PTSD highly recommend EMDR as the primary treatment option. These guidelines have been published by reputable organizations such as the World Health Organization (5), the National Institute for Health and Clinical Excellence (6), the International Society of Traumatic Stress Studies (7), and the U.S. Department of Veterans Affairs (VA) and Department of Defense (8).

EMDR therapy, developed by Shapiro (9), is a highly effective treatment for individuals suffering from post-traumatic stress disorder (PTSD). Systematic reviews and meta-analyses indicate EMDR can be more effective than control groups and comparable to other active treatments for PTSD (see, 10–13). It is based on the Adaptive Information Processing model (AIP Model, 1995) (14).

The AIP model postulates the existence of an inherent neurobiological function present in every human being, enabling them to process information. This implies that incoming information is skillfully integrated and synthesized, enhancing one’s past experiences and knowledge (15). When the traumatic event is overcome, the information is stored in a “memory network system,” and the processing and reprocessing of this information creates new associations necessary for adaptively resolving the learning of the experience. If, on the other hand, the experience is not adequately processed (as in the case of traumatic memories), information processing does not occur functionally, and the information remains isolated in the individual nodes of the neural network. Therefore, the ability to integrate information is compromised.

Consequently, it remains stored in the same way as it was experienced at the moment of the experience, with the same emotions, thoughts, beliefs, and physical sensations as at the time of the event. These aspects are not just reactions to the events that occurred but are manifestations linked to the perception of memories that have been memorized (16). A comprehensive meta-analysis conducted A.L.E. on the efficacy of EMDR therapy in treating PTSD revealed significant patterns of neural activation in various brain regions. This meta-analysis confirmed the involvement of bilateral anterior cingulate cortex (ACC), insula, inferior frontal gyrus (IFG), left precentral and cingulate gyri, and claustrum, along with the right middle and superior frontal gyri, inferior parietal lobule, globus pallidus, and thalamus. Such neurobiological evidence solidifies the foundation for a biologically grounded therapeutic approach to PTSD (17). Harnett et al. (18) confirmed these results, identifying an overlapping brain network involved in fear learning and memory, including the prefrontal cortex, hippocampus, and amygdala. This network assumes a pivotal role in the pathology of PTSD. Notably, alterations in the network’s function, structure, and biochemistry seem to underpin the cognitive-affective impairments observed in individuals with PTSD. Thus, these symptoms stem from past traumatic experiences that persistently cause distress, primarily due to inadequate processing of memories. When triggered, these stored elements cause symptoms of PTSD and other disorders.

The cognitive neuroscience of attention provides important insights into potential mechanisms of trauma treatment. Research has shown that individuals with PTSD demonstrate altered attention patterns, particularly heightened vigilance toward threat-related stimuli and difficulties disengaging from threat-related information (19). These attention biases are maintained by dysfunction in neural networks involving the anterior cingulate cortex (ACC), amygdala, and prefrontal regions that regulate emotion-attention interactions (20).

Attention Bias Modification (ABM) studies have demonstrated that systematic training of attention patterns can reduce symptoms in anxiety and trauma-related disorders (21). This suggests that interventions targeting attention control mechanisms may help reorganize maladaptive information processing. The Posner paradigm specifically engages endogenous attention networks involving top-down control from prefrontal regions - the same circuits often dysregulated in trauma (22). By requiring voluntary attention shifts while maintaining central fixation, this paradigm may help strengthen attention control abilities that are typically compromised in trauma-related disorders.

Unlike other treatments, which focus on altering emotions and thoughts, EMDR therapy directly targets memory, with the goal of changing the way it is stored in the brain to reduce and eliminate problematic symptoms. EMDR employs a dual-attention technique, in which the patient remembers the traumatic event (along with associated thoughts and emotions) while focusing on an external stimulus. This typically involves following the therapist’s fingers, moving from side to side across the visual field, thereby inducing horizontal eye movements (EM). EM sessions are repeated until the distress caused by the memory is significantly reduced, after which the patient replaces a negative memory-related thought with a positive one. EMDR’s standardized procedures, which incorporate eye movements and bilateral stimulation, appear to stimulate an accelerated learning process, reducing the vividness and emotion of the memory during therapy sessions (23).

The first scientific research providing support for the AIP model and the role of eye movement in EMDR came from the experimental research of Christman and colleagues (24, 25) through testing their hypothesis of interhemispheric interaction, suggesting that increased interaction between the two hemispheres of the brain reflects adaptive information processing. However, direct measurements of interhemispheric interaction using electroencephalography (EEG) data have cast doubt on increased interhemispheric interaction as a neurobiological mechanism underlying EMDR (26). A later study by Parker and Dagnall (27) showed that the effect of increased accessibility was stronger for horizontal eye movements than for vertical eye movements and fixation on a specific point in the room.

A frequently proposed theory suggests that when a person’s attention is drawn to a new stimulus, dual attention stimulation can trigger an orientation response. This response is a natural reaction of interest and attention. Kuiken, Bears, Miall & Smith (28) conducted a study to test this hypothesis and found that the eye movement condition was associated with improved attentional flexibility.

Barrowcliff et al. (29) suggest that in EMDR therapy, the process of orientation functions as an “investigative reflex,” triggering a relaxation response once it’s determined that there is no threat. This relaxation aids the therapeutic outcome through reciprocal inhibition. Another interpretation by Van den Hout and colleagues (30, 31) proposes that EMDR alters somatic perceptions during recovery, which disrupts the reconsolidation of traumatic memories, leading to reduced emotional distress. Stickgold (32) suggests that the orientation response triggers processes similar to those during REM sleep, activating neurobiological mechanisms that aid in consolidating episodic memories into cortical semantic memory.

However, EMDR therapy incorporates not only EM but also other bilateral stimulation (BLS), such as tones or taps, in accordance with the patient’s clinical history.

Although the success of EMDR in the treatment of trauma is widely acknowledged in the clinical field, it has also been the subject of debate (33). One aspect that has sparked controversy is using bilateral stimulation during EMDR. However, a meta-analysis suggests that bilateral stimulation can be seen as a form of distraction that engages the client’s attention, which is crucial for the effectiveness of EMDR treatment (34). Furthermore, studies have also found that other forms of stimulation, such as drawing a figure (35) and playing the computer game Tetris (36), can also have positive effects on the treatment process. These findings highlight the diverse range of options available to therapists when facilitating effective EMDR sessions. However, therapists may find that EMDR is only partially effective or ineffective for some patients (37). This could be due to using the wrong type of BLS, which can hinder progress. The choice of BLS should be based on the client’s history and characteristics; for example, if the client has a visual cognitive style (i.e., how individuals process and interpret information, affecting their cognitive process of reasoning, attention and memory recall. Those with a visual style excel in tasks involving spatial reasoning and prefer visual aids. This contrasts with verbal or analytical styles that rely more on verbal information); using visual BLS might be more effective. Sometimes, clients may not fully engage in the process or feel like it is a waste of time. A willing and open mindset allows clients to fully benefit from the therapy (38, 39). In fact, some studies demonstrate that a willing and open mindset, also described as a “growth mindset,” or the belief that personal attributes and emotions can change, can positively influence therapy outcomes. Meta-analyses show that individuals with growth mindsets experience less psychological distress, place greater value on treatment, and are more likely to engage in active coping strategies, though these effects are generally modest (39, 40, 41). While direct research on how mindset specifically affects the efficacy of EMDR is limited, related studies suggest that psychological factors such as openness, willingness, and mindfulness can influence therapeutic outcomes. Both EMDR and mindfulness therapies involve tasks that help lessen the vividness of distressing memories. An engaged mindset can improve client participation and therapeutic outcomes. (42).

Moreover, therapists often find themselves managing patients who poorly tolerate BLS by responding derisively and not accepting the treatment. To determine if the EMDR technique can be effective without EM and other BLS, we can explore other dual attention tasks. These tasks would involve the patient focusing on a cognitive task while recalling the memory. This alternative method may be helpful for patients who find the traditional method too distressing. In this regard, we have introduced a classic Posner paradigm (43) in which the client is prompted to covertly redirect his/her attention to stimuli displayed on the left and right sides of the screen, all while maintaining focus on a fixation point positioned at the center of the screen. Stimuli are displayed one by one on both sides of the screen, but the target stimulus can appear multiple times on the same side. The stimulation used in this method is different from traditional stimulation. However, the client still actively participates in an attention task that creates a cognitive load similar to traditional EMDR. The Posner paradigm represents a powerful tool for studying endogenous focused attention and the allocation of cognitive resources. It requires individuals to perform attentional shifts, directing their focus to specific points while inhibiting attention to irrelevant inputs. This shift of attention is crucial for efficiently processing information from different stimuli, but it also comes with costs. Task switching can influence performance as it demands increased effort in shifting attention.

Our aim was to explore whether an endogenous attention task (specifically, the Posner paradigm), which involves shifting spatial attention without eye movements, could be equally effective as eye movements in processing negative memories of moderate to highly traumatic intensity and provide insights into the underlying mechanisms of the technique.

We are examining the overall effects of EMDR therapy on Subjective Units of Distress (SUDs), Impact of Event Scale-Revised (IES-R), and Posttraumatic Stress Disorder Checklist for DSM-5 (PCL-5) measures. This involves comparing the effectiveness of the traditional method with EM and BLS to an alternative approach that uses an internal visuospatial attention paradigm (Posner) with the presentation of bilateral but not simultaneous stimuli.

This comparison intentionally ignores other important components of the EMDR protocol that enhance its effectiveness and focuses only on eye movements and bilateral attention. However, we continue to uphold the essential components of the EMDR protocol, such as emotional activation, the therapeutic alliance, and the process of meaning-making.

## 2 Materials and methods

### 2.1 Participants

Given the exploratory nature of our research and the aim of detecting differences between experimental groups, we conducted an *a priori* power analysis using the G*Power software (44). A conservative moderate effect size (f = 0.25) was adopted, consistent with available literature. Research on the effectiveness of EMDR in non-clinical intervention contexts is limited, with small reported effects (Cohen’s d = 0.29; 45). Regarding the Posner task in intervention scenarios, only a few studies have reported effect sizes (η²) (e.g., 46; 47), indicating that attentional cueing effects are moderate and variable. Based on this estimation, a minimum of 34 participants would be required to achieve a power of 0.80 with α = 0.05 (two-tailed) for mixed within-between ANOVAs.

**Figure 1:**
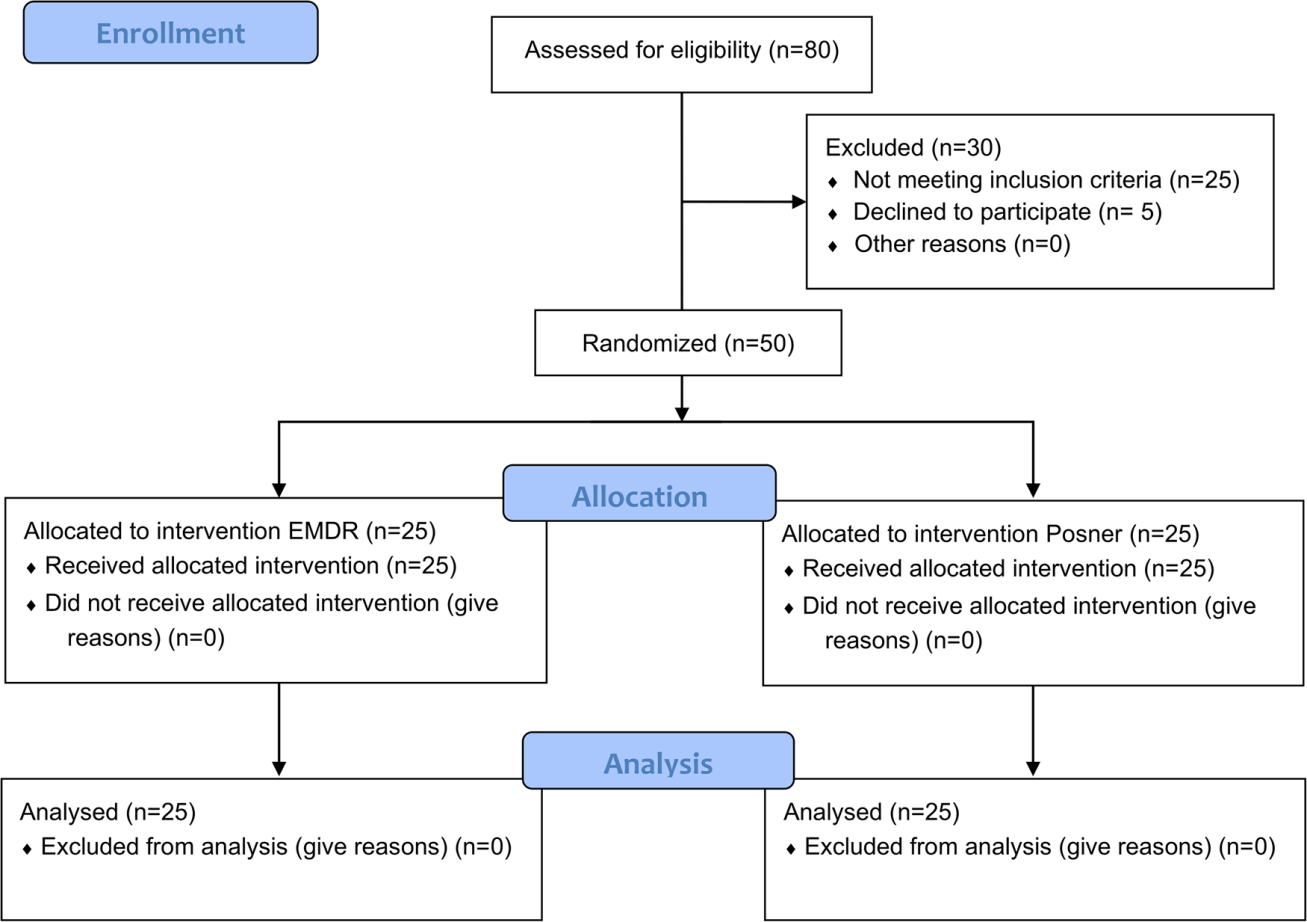
CONSORT-style flow diagram of participant progression through the study. A total of 80 individuals were assessed for eligibility. Thirty were excluded (25 did not meet inclusion criteria, 5 declined to participate). Fifty participants were randomized and allocated to either the EMDR group (n = 25) or the Posner paradigm group (n = 25). All participants received the assigned intervention and were included in the final analyses.

A total of 80 individuals were assessed for eligibility. Thirty were excluded (25 did not meet inclusion criteria, 5 declined to participate), leaving 50 participants who were randomized to the two conditions. Twenty-five were allocated to the EMDR group and 25 to the Posner paradigm group; all received the assigned intervention and were included in the final analyses (see CONSORT Flow Diagram; **Figure 1**). Participants (22 males, 28 females) were aged between 18 and 39 years (M = 24.2, SD = 3.8). No significant baseline differences were observed between groups on clinical measures: SUDs (p = 0.40), IES-R (p = 0.88), and PCL-5 (p = 0.89).

Exclusion criteria included current or past use of psychiatric medication, substance abuse, prior psychiatric treatment or hospitalization, and neurological conditions affecting the central nervous system (e.g., stroke or seizure disorder). Additionally, participants completed the Working Memory Questionnaire (WMQ; 48), a 30-item self-report instrument assessing three domains of working memory: short-term storage, attention, and executive functioning. Each domain comprises 10 items rated on a 5-point Likert scale (maximum total score = 120; maximum domain score = 40). Higher scores indicate greater difficulties. Only the short-term storage domain was used as an inclusion criterion, with a cut-off of 19. All participants scored below this threshold.

A detailed overview of participant flow, including eligibility assessment, randomization, allocation to conditions, and final inclusion, is provided in the CONSORT-style flow diagram (**Figure 1**).

All participants provided written informed consent. The study procedures were conducted in accordance with the Declaration of Helsinki and were approved by the Ethics Committee of the Department of Psychology, Sapienza University of Rome (protocol no. 0002415, December 19, 2019).

### 2.2 Experimental procedure

As a first step, all interested individual and potential participants received a link enabling them to complete several socio-anamnestic questionnaires anonymously. While this process was required for evaluating the inclusion/exclusion criteria, it also allowed the participants to provide a list of ten potentially highly stressful memories, one of which was selected as the focus of the ensuing sessions.

The specific memory to be processed was chosen for each participant by the EMDR therapist according to the following criteria:

1. It should have evoked a level of Subjective Units of Distress (SUDs; 49, 50) between 7 and 10. Specifically, SUDs is a scale used to measure the intensity of distress or discomfort that an individual feels in response to a specific situation, thought, or memory. The scale typically ranges from 0 to 10, where 0 represents no distress at all (completely relaxed or calm) and 10 represents the highest level of distress imaginable (extremely anxious or overwhelmed). SUDs are widely used and valued for their simplicity and practical application, even if formal evidence of their reliability (e.g., internal consistency) is lacking due to their single-item nature; therefore, SUDs should be interpreted within the broader clinical context, along with other scales and evidence (i.e., 51).
2. The memory chosen should be one that can be effectively addressed within three sessions. Thus, memories with significant relational components, such as attachment issues with parents or relational difficulties linked to a personality disorder, were not considered.
3. Stressful memories can be categorized into different emotional areas such as loss, unresolved grief, fears related to stressful events experienced directly or indirectly, and situations in which the person felt genuinely endangered in the past.

In order to safeguard the well-being of the participants, we deliberately refrained from addressing memories that could not be adequately processed within the time constraints of the sessions. We understand the potential beneficial impact of the treatment proposed in the study, especially when considering the effect on memories. It is important to mention that we had to limit the number of sessions to prioritize scientific accuracy and the replicability of the study. Furthermore, we provided all participants with the opportunity to be supported for up to five sessions in total, including two additional sessions after the initially planned three. This was to ensure that participants’ needs for further support in alleviating the impact of their distressing memories were fully met.

In a second step all enrolled participants were randomly assigned to one of the two experimental groups (EMDR, Posner). Each group included 25 participants. The groups differed in age (EMDR: 23.08; s.d.: 4.3 *vs*. Posner: 25.36; s.d.: 2.9; t_48_= -2.1, p = 0.03, Cohen d = -0.61) but not in gender (EMDR: 10M, 15F *vs*. Posner: 12M, 13F; p = 0.56).

Throughout all the sessions, great emphasis was placed on the organization of the setting, which was meticulously designed to recreate a cozy and inviting environment that is commonly found in psychotherapy sessions.

During the EMDR treatment, the participant is positioned on a chair, facing the therapist, and slightly shifted to the right to facilitate eye movement as per the EMDR protocol. In the Posner protocol, the participant looks at a computer showing the Posner paradigm. The therapist sits close by to make sure the participant stays focused on the screen and doesn’t move their eyes.

Afterward, the participants were contacted to schedule three 45-minute sessions. It was explained to them that each session must be completed within seven days of the previous one, as per the experimental design. This rule was established to ensure that the parameters and clinical guidelines for memory processing, as outlined in the standard EMDR protocol (52), were followed. Additionally, implementing this criterion allowed us to recreate a therapeutic environment closely aligned with a typical individual psychotherapy plan.

### 2.3 Treatments procedure

#### 2.3.1 First session

The first session focused on discussing and addressing the participant’s highly stressful memory. Treatment starts by asking about personal and family history (Phase 1). This verification process ensures that the selected memory meets the inclusion criteria and is suitable for further treatment. After selecting the memory and confirming its compatibility with the experiment, the corresponding SUDs is assessed.

Upon selecting the memory, the participant completed the Impact of Event Scale-Revised (IES-R) questionnaire online via a link (53), a standardized psychometric scale consisting of 22 items to assess the presence of post-traumatic symptoms. This self-administered instrument consists of three subscales: Re-experiencing, Hyperarousal, and Avoidance. Respondents must rate each item on a scale from 0 (not at all) to 4 (extremely), based on their experience of the traumatic event in the past 7 days. The IES-R has demonstrated strong reliability and validity across diverse populations and languages, making it a valuable tool for both clinical screening and research (54, 55). Internal consistency, as measured by Cronbach’s alpha, is consistently high for the total scale (typically 0.81–0.93) and for its subscales— Re-experiencing, Hyperarousal, and Avoidance —which generally range from 0.74 to 0.88 (e.g., 56, 57). Additionally, the Posttraumatic Stress Disorder Checklist for DSM-5 (PCL-5) (58). It is a 20-item self-report measure to assess the 20 symptoms described by the DSM-5 for PTSD, each corresponding to a specific PTSD symptom. Participants are asked to respond on a Likert scale, ranging from 0 (not at all) to 4 (extremely). The PCL-5 consistently demonstrates excellent reliability across diverse populations and settings. Internal consistency is very high, with Cronbach’s alpha values typically ranging from 0.94 to 0.96 in both clinical and non-clinical samples (59, 60). Subscales also show strong internal consistency, with alphas above 0.79 (61). Test-retest reliability is robust, with coefficients between 0.82 and 0.89 over intervals of several days to weeks (59).

After filling out the two questionnaires from the standard EMDR protocol, participants are taught about EMDR, which includes a “Stop” signal for pausing the BLS for the first group or the Posner protocol for the second group. Then, the preparation phase (Phase 2) can begin. Participants were encouraged to select a safe place that made them feel comfortable and positive. For the participants in the group receiving traditional EMDR therapy, the safe place was strengthened through a brief and slow series of EM.

For the Posner group, the safe place was installed in a similar way as outlined in the EMDR protocol. However, they did not use any EM to strengthen the mental image of the safe place. Participants in both groups were asked to choose a keyword to associate with their safe place, like forest, sea, mountain, serenity, etc. They were then instructed to create a mental connection between this keyword and their chosen safe place. In the group undergoing eye movement stimulation, the association with the keyword was consistently strengthened through a brief and slow series of EM. After installing the safe place, we proceeded to Phase 3 of the standard EMDR protocol, which involved the assessment phase. In this phase, participants were instructed to recall the unpleasant memory. They were directed to identify the specific image that represented the most distressing aspect of the memory by answering the following question: “*When you think about that memory what picture or image represents the worst or most powerful part? What do you visualize?*” Additionally, the participant was asked to state the negative belief associated with the memory, using the question: “ *When you think about that memory or image, what negative belief do you have about yourself now?*” Negative thoughts are categorized into three main areas: responsibility (highlighting both self-defectiveness and guilt), safety, and control over choices. Similarly, each participant was also asked to indicate positive beliefs with the following question: “ *When you bring up the memory, image, or incident, what would you like to believe about yourself now?*” Also positive beliefs were categorized into three main areas: responsibility (divided into self-defectiveness and guilt), safety, and choice control. Once the negative and positive beliefs were identified, we assessed the Validity of the Positive Cognition (VoC), namely, how much the participant felt to believe in the positive cognition with respect to the unpleasant memory. The question asked was: “*When you think of that memory or image, how true does (repeat the positive cognition) feel to you now on a scale of 1 to 7 where 1 feels completely false and 7 feels completely true?*”

At this stage, we identified the emotions associated with the memory. We also assessed the SUDs level and identified the point in the body where the participant felt a disturbance related to the memory. In the group receiving Posner stimulation, we conducted a short training session to familiarize the participants with the Posner experimental paradigm. This involved reading the instructions displayed on the screen and performing a few training trials (i.e., 24), during which participants were explicitly trained to orient their attention without shifting their gaze from central fixation. At the end of the initial session, each participant was prompted to recall a safe place where they could find calm before leaving the therapeutic environment.

#### 2.3.2 Second session

In the second session, for both groups, the reprocessing of the unpleasant memory took place. Each participant was briefed with a recap of the elements that emerged in Phase 3, moreover, before starting the new session, the use of the “Stop” signal was reminded in case the processing would become too intense. Then, the initial instruction was read: “*Every so often I will do a simple check on what you are experiencing. All you need to do is tell me about what you are experiencing so I can make the proper choices. There is no right or wrong way to do EMDR. Sometimes things will change and sometimes they won’t. Just give me accurate feedback about what is happening and let whatever happens, happen. Also, remember you are the one in control, and if you need to stop, just use your stop signal.*

After assessing the level of SUDs and (negative and positive) cognitions, the desensitization phase (Phase 4) was initiated. In the group undergoing Posner stimulation, a brief additional training was conducted to ensure that the participant understood the task of the Posner paradigm and that they could perform it without moving their eyes, keeping them fixed at the center of the screen and only shifting attention on the side indicated by the arrow (see the below paragraph about Posner Task for details).

For the group undergoing EM stimulation, the initial instruction was: “*Now recall the image, those negative words*” (the therapist repeated the negative cognition) “*and notice where you feel it in your body, then follow my fingers.*” Then, sets of 25–35 stimulations were used between each stimulation.

For the Posner group, the initial instruction was similar to the latter, but in addition, the participant was asked to keep their gaze fixed at the center of the screen and only shift attention, following the instructions previously displayed. In this group, a set of experimental blocks of ~35-sec duration was prepared to closely match the range of stimulations specified in the standard EMDR protocol.

The conclusion of the second session involved for both groups, the recalling of the safe place at the end of the reprocessing through stimulation (EM or Posner). Participants were informed that processing could continue after the session and that they might notice new insights, sensations, thoughts, memories, or dreams. They were advised to take note of these experiences if they occurred and discuss them in the third session. The therapist remained available for psychological support throughout all three sessions. Finally, participants were also recommended to use a safe place to alleviate any distress or discomfort resulting from overthinking about the stressful memory.

#### 2.3.3 Third session

The third and last session started for both groups with a recap of the previous session, according to the evaluation protocol (52), remembering the previous session’s content to the participants. The participants were also asked to report if they noticed possible changes after the previous session and their current perception of the memory we were working on.

At this stage, the SUDs were re-evaluated, and if it remained different from 0, desensitization was continued; if it had dropped to 0, installation of the positive cognition was carried out, following the standard EMDR protocol. As is customary, this session concludes with a reminder of the safe place and a note about the potential for continued memory processing beyond the session.

Before leaving the room, participants were asked to complete again IES-R, PCL-5 and SUDs referring to the memory they had worked on. At the end of the third session, the therapist could add two more sessions if necessary or requested by the participant for their benefit. However, these additional sessions would not be taken into consideration for this study.

#### 2.3.4 Posner task

**Figure 2:**
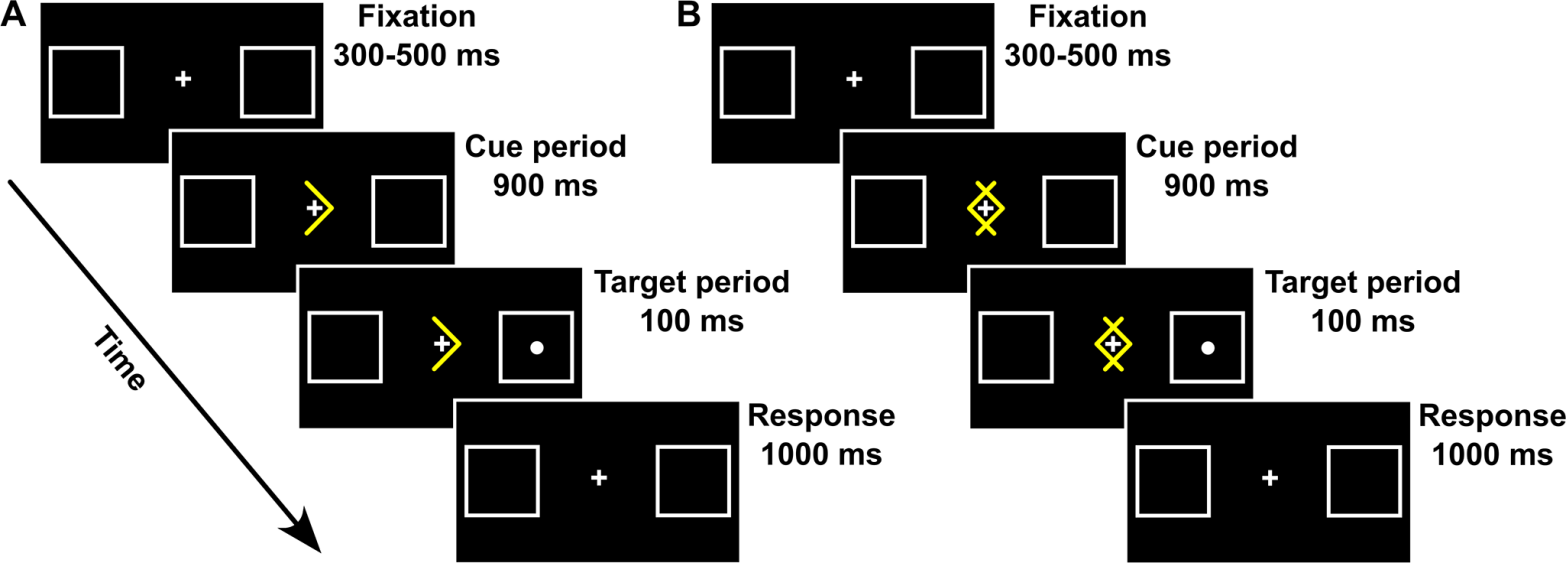
Spatial arrangement, timing, and events of directional **(A)** and non-directional **(B)** trial types used in the Posner task.

To maintain therapeutic equivalence with traditional EMDR sessions, the computerized Posner task was administered in blocks that matched the temporal structure of EMDR bilateral simulation. Each experimental blocks lasted ~35 sec (comparable to 25–35 EM stimulations in standard EMDR), with brief pauses between blocks allowing for the same therapeutic check-ins used in conventional EMDR (i.e., asking participants about their current experience, SUDs level, and any emerging thoughts or sensations). The total number of blocks administered was determined by the same clinical criteria used in traditional EMDR, namely, when participants reported a significant reduction in distress or when the session naturally concluded after reprocessing. Each block of the Posner task included 15 trials (8 Valid, 2 Invalid, 2 Neutral, and 3 Catch trials). Each trial began with a “*Fixation*” period lasting 300–500 ms (uniform distribution), in which a fixation cross (size: 1° x 1°) was presented at a central position together with two lateral boxes (size: 4° x 4°), one centered 7.5° to the left and the other 7.5° to the right (see **Figure 2**). All stimuli were white against a black background. The “*Fixation*” period was followed by a “Cue” period, lasting 900 ms. At the beginning of the “*Cue*” period, a highly predictive (80%) yellow arrow (size: 2° x 3°) was presented around the central fixation cross. On directional Valid and Invalid trials, arrows pointed to the right or to the left side of space, and participants were instructed to covertly pay attention to the box indicated by the arrow, avoiding eye movements. On nondirectional Neutral trials, two overlapping yellow arrows, pointing to both the left and right sides of space were presented (see **Figure 2**), and participants were instructed that, in this case, they did not have to orient their attention to one of the two boxes before target occurrence. At the end of the “*Cue*” period, a white dot (size: 0.5◦ × 0.5◦) was presented as a target for 100 ms at the center of one of the two boxes. Once the cue and the target disappeared, 1000 ms were allowed for response collection (“*Response*” period). Participants were required to maintain their gaze on the central fixation point throughout the trial and were asked to detect the appearance of the target by pressing a button with their right index finger as soon as possible or withhold their response if no target was presented (Catch trials). On Valid trials, the target was presented in the box cued by the arrow, while on Invalid trials, the target was presented in the box as opposed to the one cued by the arrow. On Neutral trials, the target was presented with equal probability in one of the two boxes. In Catch trials, no target followed the cue presentation. For the Posner group, the participant was asked to keep their gaze fixed at the center of the screen and only shift their attention, following the instructions previously displayed, to perform the task. Prior to starting the experimental procedure, all participants performed a practice block of 15 trials. Each participant performed a variable number of experimental blocks, with a minimum of 7 and a maximum of 34 blocks (mean 19.6 blocks).

### 2.4 Statistical analysis

#### 2.4.1 Posner task

Prior to statistical analysis for testing the attentional performance obtained in the Posner task, all RTs exceeding 2 standard deviations around the experimental group’s mean were considered outliers. This procedure resulted in the exclusion of less than 2% of responses in each trial type. Successively, individual mean RTs were entered in a repeated measures ANOVA with factors Session (Second, Third) and Trial type (Valid, Neutral, Invalid). Importantly, since the number of administered blocks varied among participants, the individual total number of trials was considered as a covariate in the ANOVA. Eventual significant main effects and interactions were further explored using Bonferroni *post-hoc* comparisons.

#### 2.4.2 Treatment effectiveness

Scores to SUDs, PCL-5, and IES-R clinical indexes were considered for the analysis of treatment effectiveness. In the case of IES-R, both the total score and scores to the different subscales (i.e., Re-experiencing, Hyperarousal, and Avoidance) were considered. More specifically, for each of these dependent variables, we fitted two complementary series of linear mixed-effects models.

In the first set of models, we evaluate the overall pre–post symptom reduction, as well as the possibility that such changes were differentially modulated by Age across treatments. We thus included Time (pre, post) as a within-subject factor, Treatment (EMDR *vs*. Posner) as a between-subject factor, and Age as a continuous predictor. All main effects and their interactions (Time × Treatment, Time × Age, Treatment × Age, and Time × Treatment × Age) were specified as fixed effects. A random intercept for participants was included to account for repeated measurements. Tests of fixed effects were based on Type III sums of squares.

The second set of models was instead adopted to assess whether treatment-related symptom changes differed by sex (male, female) that was included as a categorical between-subject factor in place of Age. This resulted in 2 (Time: pre, post) × 2 (Treatment: EMDR, Posner) × 2 (Sex: male, female) mixed models, with random intercepts for participants. Again, all main effects and interactions (Time × Treatment, Time × Sex, Treatment × Sex, and the three-way interaction Time × Treatment × Sex) were tested using Type III sums of squares.

All statistical analyses were conducted in Jamovi (version 2.6.44), using the GAMLj3 module for linear mixed models.

As a further step, we used the TOSTER add-on of Jamovi (62) to run equivalence analysis in order to determine whether Posner effectiveness could be considered equivalent to EMDR (63, 64). First, for SUDs, IES-R, and PCL-5 scores effectiveness of our treatment was computed. This was calculated at the individual level as the mean difference between Post and Pre sessions. For all our measures, the null hypothesis is (Posner – EMDR) ≥ δ, and the alternative hypothesis is (Posner – EMDR) < δ, where δ is the margin set at the minimal clinical relevance (65). If both the upper and lower bounds of the 90% confidence interval lie entirely within the range defined by ±δ, then it could be concluded that Posner is equivalent to EMDR. For SUDs the margin was δ = 3, while for IES-R it was equal to δ = 10, and for PCL-5 the margin was δ = 1. These were determined by clinical experts based on consensus and practical experience, as well as a deep reviewing of the existing guidelines and empirical data that helped them to define what constitutes a significant change on these scales (e.g., 66 for IES-R; 67–69).To verify whether the sample size, initially determined by testing the differences between experimental groups, still provided sufficient statistical power for TOST, we conducted a *post-hoc* power analysis. Here, we used the equivalence bounds, along with the observed pooled standard deviations of the change scores (Post – Pre). The resulting power was 99.9% for both SUDs and IES-R, confirming that our sample size was adequate to detect equivalence within these margins. Regarding PCL-5, the *post-hoc* power analysis yielded a statistical power of 76.8%, which is slightly below the conventional threshold of 80%. For this reason, we recommend taking the conclusion of this index with caution (see the limitations section).

Finally, following Jacobson and Truax (70), we calculated the Reliable Change Index (RCI) to determine whether the pre–post differences exceeded what would be expected due to measurement error. Using the baseline SDs from our sample and published test–retest reliabilities, reliable change thresholds were estimated at 17.5 points for the PCL-5 and 13.5 points for the IES-R. Specifically, for the PCL-5, we relied on the test–retest reliability reported by Wortmann et al. (60; rtt = .84) and for the IES-R on the value reported by Creamer, Bell, and Failla (66; rtt = .91).

## 3 Results

### 3.1 Posner task

**Figure 3:**
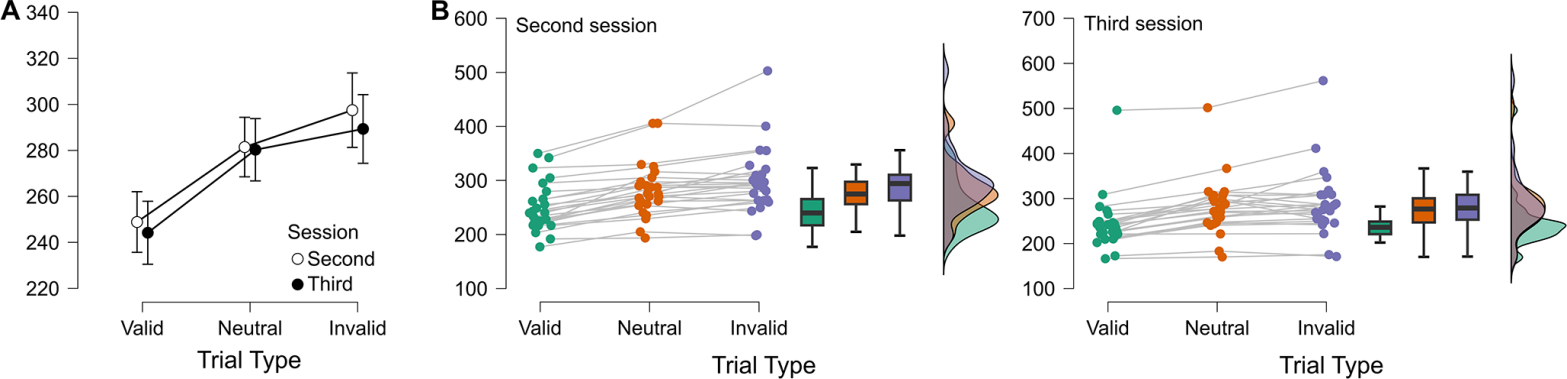
**(A)** Mean RTs with standard errors are shown as a function of trial type (Valid, Neutral, Invalid) and session (Second *vs*. Third). **(B)** Individual RT distributions for the Second (left) and Third (right) sessions. Colored dots indicate single-subject values for each trial type (green = Valid, orange = Neutral, violet = Invalid), with lines connecting repeated measures from the same participant. Boxplots summarize the central tendency and dispersion, while violin plots depict the density distribution of scores.

In line with conventional findings from the Posner task (22) our participants showed a significant validity effect with faster RTs to Valid (246.5 ms) as compared to Invalid (293.4, p <.001) targets (F_(2,48)_ = 63.1, p <.001, η^2p^ = .72; See **Figure 3**). Attentional benefits (Bonferroni *post-hoc* comparison: RTs difference between Valid and Neutral trials; 34.3 ms, p <.001) and Costs (Bonferroni *post-hoc* comparison: RTs difference between Invalid and Neutral trials; 13.4 ms, p = .01) were also significant (see **Figure 3**). No main effect or interactions were found between the Session and the total number of trials (both p >.2), indicating no difference in the performance on the Posner task between the second and third sessions or as a function of the number of administered trials.

### 3.2 Treatment effectiveness

**Figure 4:**
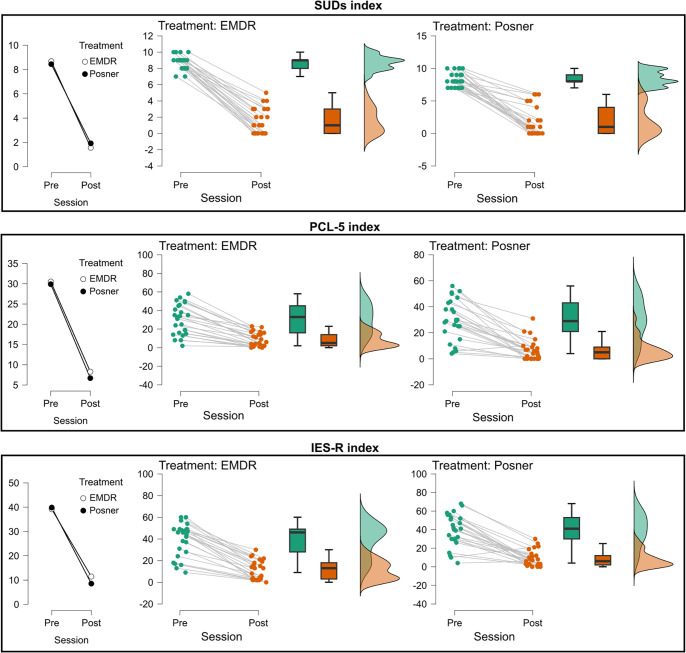
Changes in subjective distress and post-traumatic symptomatology across treatment sessions. Mean values (left plots), individual trajectories (middle plots), and score distributions (right plots) are shown for the three clinical measures: SUDs index (top), PCL-5 index (middle), and IES-R total index (bottom). For each treatment (EMDR, Posner), data are presented at Pre- and Post-session. Treatments are represented by different markers (EMDR = white circles, Posner = black circles). Middle panels report individual data points (green = Pre, orange = Post) with connecting lines indicating within-subject changes, boxplots summarizing the group distribution, and violin plots on the right illustrating the overall score distribution.

For SUDs, there was a strong main effect of Time (F(1,47) = 634.22, p <.001; Estimate = 6.82, t_47_ = 25.18), with scores significantly lower at post compared to pre (see **Figure 4**). Age was also significant (F(1,46) = 6.35, p = .015; Estimate = 0.12, t_46_ = 2.52) and the Age × Time interaction was significant, (F(1,47) = 6.15, p = .017; Estimate = -0.18, t_47_ = -2.48), indicating that older participants showed smaller reductions. No significant effects were found for Treatment (F(1,46) = 0.40, p = .53) for Time × Treatment (F(1,47) = 0.1, p = .75), or for Age × Treatment (F(1,46) = 0.70, p = .41).

For the PCL-5, a robust main effect of Time was observed (F(1,47) = 149.42, p <.001; Estimate = 22.72, t_47_ = 12.22) with lower scores at post (see **Figure 4**). Age was not significant, (F(1,46) = 0.000236, p = .98), nor was the Age × Time interaction (F(1,47) = 0.0012, p = .97). No main effect of Treatment (F(1,46) = 0.13, p = .72), or Time × Treatment interaction (F(1,47) = 0.06, p = .81), was found. However, the Age × Treatment interaction reached significance (F(1,46) = 4.29, p = .044; Estimate = 1.81, t_46_ = 2.07), indicating differential age-related effects between groups.

For the IES-R total score, there was a strong main effect of Time (F(1,47) = 206.48, p <.001; Estimate = 29.52, t_47_ = 14.37; (see **Figure 4**). Age was not significant (F(1,46) = 0.13, p = .72) as well as Treatment (F(1,46) = 0.053, p = .82). Similarly, no significant two-way interactions were found (Age × Time: F(1,47) = 0.83, p = .37; Age × Treatment: F(1,46) = 3.83, p = .057; Time × Treatment F(1,47) = 1.23, p = .27).

**Figure 5:**
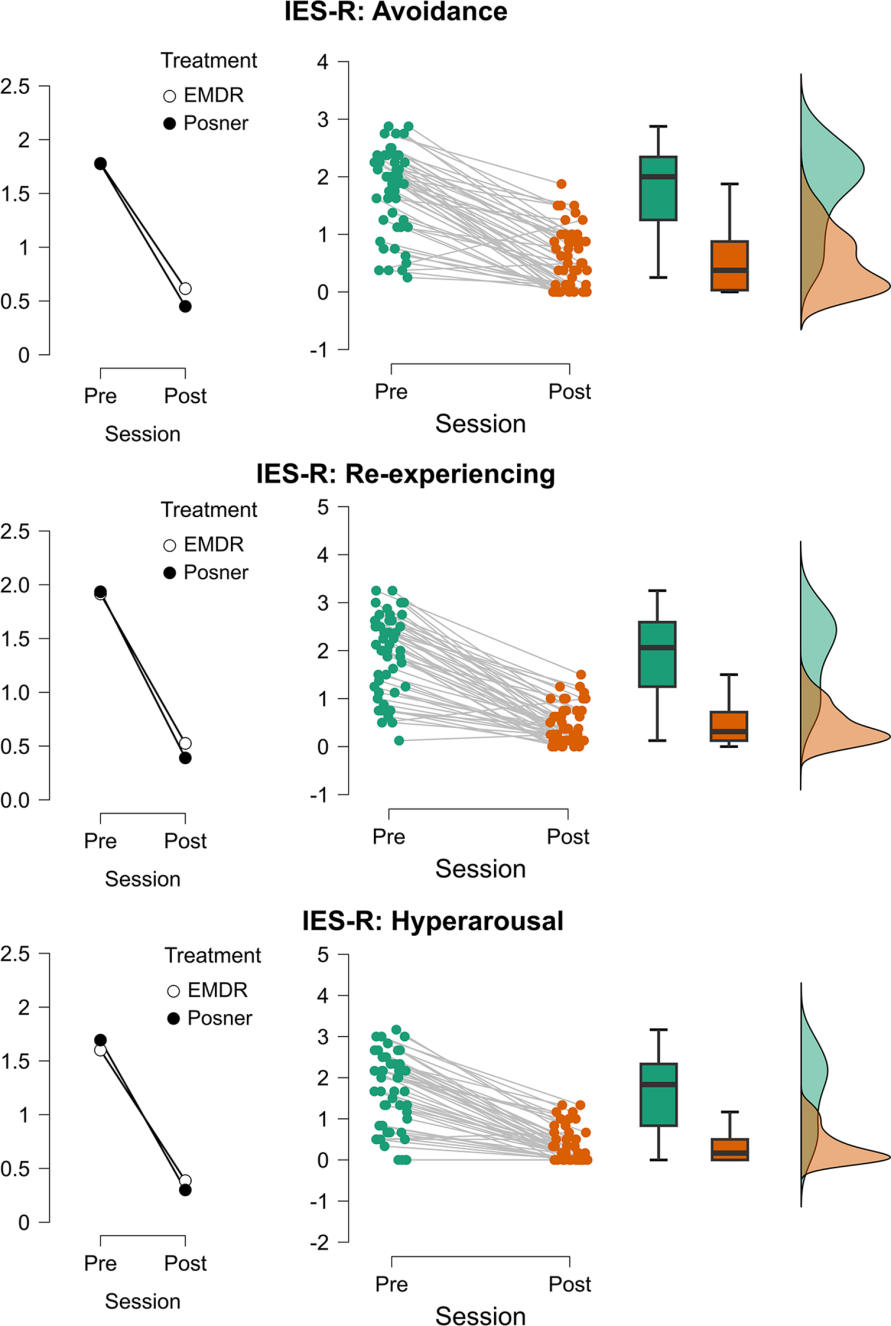
Changes in post-traumatic stress symptom dimensions across treatment sessions. Mean values (left plots), individual trajectories (center plots), and distribution densities (right plots) of IES-R subscales are displayed separately for Avoidance (top), Re-experiencing (middle), and Hyperarousal (bottom). Scores are shown for each participant before (Pre) and after (Post) treatment sessions. Treatments are represented by different markers (EMDR = white circles, Posner = black circles). Middle panels report individual data points (green = Pre, orange = Post) with connecting lines indicating within-subject changes, boxplots summarizing the group distribution, and violin plots on the right illustrating the overall score distribution.

Analyses on the IES-R subscales (Re-experiencing, Hyperarousal, and Avoidance; see (see **Figure 5**; **Supplementary Material** for complete report of the models’ output) consistently revealed strong main effects of Time (all Fs > 139.8, all ps <.001), with significant reductions from pre- to post-assessment (see **Figure 5**). No main effects of Treatment or Age were detected across the three subscales (all ps ≥.28), and interactions involving Time were not significant (all ps ≥.18). Importantly, for the Re-experiencing subscale, the Age × Treatment interaction was significant (F(1,46) = 4.65, p = .036; Estimate = 0.093, t_46_ = 2.16), indicating that symptom expression varied with age across groups.

As far as the models accounting for Sex as a factor, in line with the first series of analyses, all revealed large main effects of Time (ps <.001), confirming substantial symptom reductions from pre- to post-assessment across SUDs, PCL-5, and IES-R (total and subscales). Importantly, neither Treatment nor Sex showed significant main effects, and critically, no interaction involving Sex was significant, indicating that reductions were comparable across males and females in both treatment conditions (see **Supplementary Material** for complete report of the models’ output).

**Figure 6:**
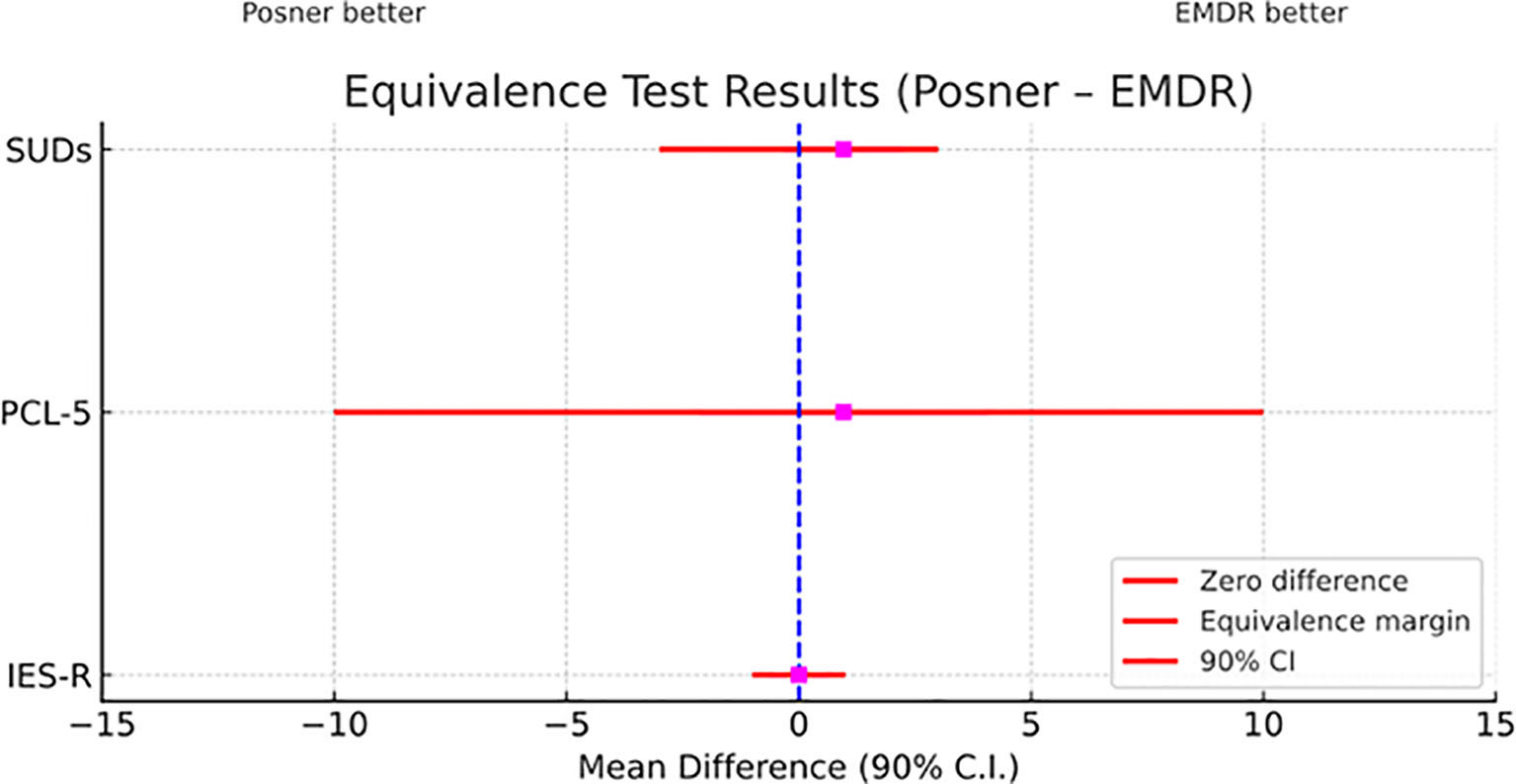
Equivalence test comparing EMDR and Posner treatments across clinical measures. Mean differences (Posner – EMDR) with 90% confidence intervals are shown for the three indices (SUDs, PCL-5, IES-R). The dashed vertical line marks zero difference between treatments, while red horizontal lines indicate the predefined equivalence margins. CIs entirely contained within these margins support statistical equivalence between the two treatments.

Most importantly, as pointed out by TOST procedure, scores of SUDs, PCL-5, and IES-R showed equivalence of Posner to EMDR (see **Table 1** and **Figure 6**). Moreover, according to the RCI analysis, 65% of participants showed reliable improvement on the PCL-5 and 88% on the IES-R. For the SUDs, no reliable change index could be computed due to the absence of published test–retest reliability; therefore, results are reported descriptively.

**Table 1:** The table reports the results of the equivalence analysis (TOST).

Equivalence results		t	df	p
SUDs	t-test	1.05	38.4	= .299
	TOST Upper	6.28	38.4	**< .001**
	TOST Lower	-4.17	38.4	**< .001**
PCL-5	t-test	-0.261	46.6	= .795
	TOST Upper	2.46	46.6	**= .009**
	TOST Lower	-2.98	46.6	**= .002**
IES-R	t-test	-0.878	40.9	= .385
	TOST Upper	3.10	40.9	**= .002**
	TOST Lower	-4.86	40.9	**< .001**

The t-test refers to a standard independent-samples t-test on post–pre difference scores. The TOST Lower and TOST Upper rows represent the two one-sided tests used to assess whether the group difference lies entirely within the pre-specified equivalence bounds. Statistical equivalence is supported when both one-sided tests are significant (71).Bold values highlight significant p values.

## 4 Discussion

In the present study, we aimed to assess the comprehensive impact of EMDR therapy on SUDs, IES-R, and PCL-5 measures by comparing the effectiveness of the traditional method with EM and BLS to an alternative approach using an endogenous visuospatial attention paradigm (i.e., Posner task), with bilateral but not simultaneous stimuli’s presentation.

### 4.1 Clinical effectiveness and mechanisms

Our results demonstrate that both conventional EMDR and the Posner paradigm produced significant and equivalent reductions in distress measures across all assessed indices (SUDs, IES-R, and PCL-5). This equivalence suggests that the therapeutic mechanism underlying EMDR may be more fundamentally related to attentional engagement than to the specific modality of bilateral stimulation itself.

Our findings align with emerging cognitive neuroscience research on attention’s role in emotional regulation and memory processing. The equivalence between EMDR and Posner paradigm effects suggests that strengthening attention control networks may be a key mechanism underlying trauma treatment. Recent neuroimaging studies have shown that successful EMDR therapy is associated with enhanced activation in prefrontal regions responsible for attention control and emotional regulation (33).

The Posner paradigm specifically engages the dorsal attention network, including the frontal eye fields and superior parietal lobule, which exert top-down control over stimulus processing (72). This network shows altered functioning in PTSD (73). By systematically activating these circuits through controlled attention shifts, both EMDR and the Posner task may help restore more adaptive patterns of information processing.

This interpretation is supported by research on attention bias modification, which has shown that training attention control can reduce trauma symptoms (74). Our results suggest that different methods of engaging attention control networks - whether through eye movements or covert attention shifts - may achieve similar therapeutic effects by strengthening these fundamental cognitive control mechanisms. This has important implications for developing alternative trauma treatments that target attention processes.

From a clinical perspective, this finding has important implications. The Posner paradigm requires participants to engage endogenous (top-down) attention mechanisms, receiving centrally presented symbolic cues (arrows) that demand voluntary, goal-directed shifts of attention based on internal interpretation. This cognitive engagement may facilitate memory reprocessing through sustained attentional load rather than through the rhythmic bilateral stimulation traditionally emphasized in EMDR protocols.

According to Shapiro (9), there are two potential interpretations for the effects of alternating bilateral stimulation. Firstly, it may enhance the processing of any emotionally charged material in general. Secondly, it may specifically target the reintegration of fragmented information related to traumatic experiences. Importantly, clinicians have also reported a reduction in the intensity and arousal associated with trauma-related stimuli following EMDR therapy. Furthermore, neuroimaging studies have demonstrated a decrease in the activation of limbic areas and an increase in the activation of prefrontal brain regions responsible for cognitive control after successful EMDR treatments (75, 76).

Neuroimaging studies have consistently shown that EMDR therapy is effective when there is increased activation in the prefrontal cortex. This suggests that attentional mechanisms play a key role in the success of EMDR therapy. Specifically, using endogenous attention rather than exogenous attention, as in the classical Posner paradigm, could have longer-lasting effects. Indeed, the attentional dislocation effect caused by endogenous stimuli lasts longer than that caused by exogenous stimuli, and this effect also persists when the stimulation is asynchronous and not simultaneous (77).

### 4.2 Clinical applications and patient acceptability

Within this theoretical framework, we propose that the use of EM or BLS is not the only way to achieve the desired therapeutic effect. Instead, we suggest that the effect results from the participant’s internal focus of attention, which facilitates reprocessing of distressing memories. The Posner task itself is a cognitive challenge that requires the participant to maintain focus on the task without moving their eyes and to receive centrally presented, symbolic cues (such as arrows) that require voluntary, goal-directed shifts of attention based on internal interpretation, engaging endogenous (top-down) attention mechanisms. Additionally, the use of the Posner paradigm can offer practical and clinical benefits. It may enhance patient engagement, particularly for patients who find traditional BSL disturbing or dismissive. The Posner paradigm offers a more cognitively demanding alternative that may facilitate better therapeutic engagement. Some patients report that activities like following fingers or performing tapping tasks are too simple and do not allow sufficient emotional withdrawal from the unpleasant memory. Tasks requiring higher cognitive engagement, like the Posner procedure, may be easier for these patients to accept. Undoubtedly, the paradigm can be adapted for patients who may have difficulty with traditional eye movements, including those with visual impairments, neurological conditions, or cultural reservations about direct eye contact with therapists. The task provides consistent, measurable cognitive demand that can be precisely controlled and replicated across sessions, potentially offering more standardized treatment delivery. Furthermore, by maintaining the same therapeutic framework, including preparation phases, safe place installation, and regular check-ins, the Posner paradigm preserves the essential relational components of EMDR while modifying only the bilateral stimulation component.

It is essential to note that our study intentionally focused on comparing eye movements and bilateral attention while maintaining other key components of the EMDR protocol, such as emotional activation, the therapeutic alliance, and the process of meaning-making. These elements remained consistent across both treatment groups, ensuring that any differences in outcomes could be attributed to the attentional mechanism rather than to variations in overall therapeutic approach.

### 4.3 Limitations and future perspectives

#### 4.3.1 Current study limitations

This study is not without limitations. First, there is a lack of a clinical population. Our reference sample consists of a healthy population reporting a list of distressing events, with SUDs ranging from 7 to 10. However, it is not the first study to use a non-clinical sample in the EMDR field; indeed, Matthijssen et al. (67) also employed non-clinical participants to test the effects of EMDR and EMDR 2.0.

There are several benefits to using a non-clinical sample. First, it allows for isolating and examining the basic mechanisms of EMDR, without the confounding effects of comorbidities or severe psychopathology that are often present in clinical populations (e.g., 67, 78). Non-clinical samples provide a controlled environment for testing theoretical assumptions, refining protocols, and comparing different approaches to EMDR. Additionally, research with non-clinical participants is often more feasible, less resource-intensive, and can be conducted with fewer ethical concerns, making it an efficient first step in the development of interventions.

However, findings from non-clinical samples must be interpreted cautiously, as their generalizability to clinical populations is limited, and further research with clinical samples is always necessary to confirm effectiveness. Therefore, it would be necessary to replicate this study using a sample of patients with PTSD to examine the effectiveness of Posner’s paradigm. Additionally, it is noteworthy to highlight that for PCL-5, our statistical power analysis for the TOST equivalence test was slightly below the common threshold of 80%. While there is clear comparability between the two methods for SUDs and IES-R, results for PCL-5 should be interpreted with caution. The PCL-5 showed greater variability in responses, suggesting that future studies should include larger sample sizes to provide more definitive conclusions about equivalence on this measure.

Finally, while this study primarily focused on overall efficacy, future research should investigate the effects of the two treatments on specific cognitive processes, such as vividness of memories and working memory capacity. Additionally, exploring the executive functions of cognitive control and response inhibition could provide valuable insights. Evaluating the specific effects on cognitive functioning could lead to the development of cognitive training programs that can be combined with EMDR therapy, especially in cases where alterations in cognitive processes are present.

Another significant limitation is the lack of follow-up data, which makes it challenging to evaluate the persistence of the effects. Indeed, without tracking outcomes over time, we cannot determine whether therapeutic benefits endure, if there are differences in relapse rates among techniques, or if one approach offers superior long-term stability.

#### 4.3.2 Future directions

The most immediate priority is replicating these findings in clinical populations with diagnosed PTSD. Such studies would determine whether the equivalence observed in our non-clinical sample extends to individuals with more severe symptomatology and complex trauma histories. Future studies should systematically examine the specific cognitive processes underlying therapeutic change in both traditional EMDR and the Posner paradigm. This includes measuring: Changes in memory vividness and emotionality across sessions; Working memory load during different phases of treatment; Attentional control and executive function improvements; and neural correlates using neuroimaging techniques (fMRI, EEG).

The success of the Posner visual attention paradigm suggests that other sensory modalities might also be effective. Future research should explore: auditory attention tasks with non-simultaneous stimulus presentation; tactile attention paradigms for patients unable to perform visual tasks; and multimodal approaches combining different attentional demands. These alternatives would be particularly valuable for patients with visual impairments or other conditions that preclude the use of traditional eye movement protocols. The Posner paradigm offers particular advantages for teletherapy applications. Several studies (79–83) have consistently demonstrated how online psychotherapy has effectively addressed issues of accessibility and “democratic” care by reaching individuals who were previously considered “unreachable.”

The pandemic has significantly accelerated the digitalization of psychotherapy. A systematic review by Lenferink et al. (84) examined the effects of online EMDR, finding that internet-delivered combinations of CBT and EMDR successfully reduced PTSD symptoms. Additionally, several studies have demonstrated the effectiveness of online EMDR, with clinically meaningful reductions in PTSD, depression, and anxiety (85), reporting similar outcomes based on SUDs compared to previous face-to-face EMDR studies (86).

Bursnall et al. (87) conducted comprehensive surveys and interviews to explore the implementation of online EMDR therapy. The study revealed that 88% of clients expressed comfort receiving EMDR therapy through digital means. However, at the onset of social distancing, 54% of therapists harbored reservations toward delivering online EMDR therapy, a figure that decreased to just 11% within a year, speaking volumes about growing acceptability.

Without a doubt, tools like the Posner task facilitate remote client care. While this is achievable with traditional EMDR, many therapists face difficulties maintaining contact with remote clients during BLS with EM. The Posner paradigm may address this challenge as patients become more actively engaged in the computerized task, potentially improving treatment fidelity in telehealth settings.

Furthermore, it is essential to plan a comprehensive long-term follow-up study in the near future to investigate the sustained positive effects of the Posner Task compared to traditional approaches. Future research should incorporate a well-structured experimental design, featuring multiple follow-up intervals at 1, 3, 6, and 12 months. This will facilitate the assessment of relapse rates and the evaluation of functional outcomes.

## 5 Conclusion

In conclusion, our findings demonstrate that endogenous visuospatial tasks, such as those employed in the Posner paradigm, may serve as viable alternatives to traditional eye movements in EMDR therapy. The results suggest that the mechanism of attention shifting, rather than the specific modality of bilateral eye movements, plays a critical role in the therapeutic process. Furthermore, our findings indicate that the simultaneous presentation of stimuli may not be a crucial aspect of EMDR’s effectiveness. From a clinical standpoint, the Posner paradigm represents a valuable addition to the therapeutic toolkit, particularly for patients who struggle with traditional bilateral stimulation methods or for implementation in remote therapy settings. However, our findings reflect the attenuation of normal distress responses in a non-clinical sample and highlight the need for future studies to replicate our results in trauma-exposed clinical populations.

This study contributes to the understanding of EMDR by highlighting the importance of attentional processes in memory processing and opens avenues for further research into alternative therapeutic techniques that leverage cognitive mechanisms. The implications extend beyond theoretical understanding to practical applications that could enhance treatment accessibility, patient engagement, and therapeutic outcomes across diverse clinical settings. It is important to note, however, that our findings were obtained from a non-clinical sample of healthy participants recalling distress memories, and therefore, replication studies with clinical populations diagnosed with PTSD or other trauma-related disorders are essential to confirm the therapeutic efficacy of the Posner paradigm in clinical settings. However, without a follow-up, we cannot determine whether therapeutic gains are maintained, whether relapse rates differ between approaches, or whether one method shows superior long-term stability.

## Data Availability

The raw data supporting the conclusions of this article will be made available by the authors, without undue reservation.
