# Supplementary material for: Exploring EMDR: an innovative approach with Posner Paradigm to reprocessing negative memories in a non-clinical sample
Source: Front Psychiatry. 2025 Nov 24;16:1605608. doi: 10.3389/fpsyt.2025.1605608 (PMC12682879; doi:10.3389/fpsyt.2025.1605608)
Supplement: Supplementary file 1 [file Table1.docx]

**Linear Mixed Model SUDs**

| Model Info | | |
| --- | --- | --- |
| **Info** |  |  |
| Model Type | Mixed Model | Linear Mixed model for continuous y |
| Model | lmer | SUDs ~ 1 + Age + Time + Treatment + Time:Treatment + Age:Time + Age:Treatment + ( 1 \| Subjects ) |
| Distribution | Gaussian | Normal distribution of residuals |
| Direction | y | Dependend variable scores |
| Optimizer | bobyqa |  |
| DF method | Satterthwaite |  |
| Sample size | 100 |  |
| Converged | yes |  |
| Y transform | none |  |
| C.I. method | Wald |  |
| Nota. All covariates are centered to the mean | | |

**Model Results**

| Model Fit | | | | |
| --- | --- | --- | --- | --- |
| **Type** | **R²** | **df** | **LRT X²** | **p** |
| Conditional | 0.871 | 7 | 194.186 | <.001 |
| Marginal | 0.847 | 6 | 194.186 | <.001 |

| Fixed Effects Omnibus Tests | | | | |
| --- | --- | --- | --- | --- |
|  | **F** | **df** | **df (res)** | **p** |
| **Age** | 6.3450 | 1 | 46.0 | 0.015 |
| **Time** | 634.2193 | 1 | 47.0 | <.001 |
| **Treatment** | 0.3985 | 1 | 46.0 | 0.531 |
| **Time ✻ Treatment** | 0.0989 | 1 | 47.0 | 0.754 |
| **Age ✻ Time** | 6.1457 | 1 | 47.0 | 0.017 |
| **Age ✻ Treatment** | 0.7029 | 1 | 46.0 | 0.406 |

| Parameter Estimates (Fixed coefficients) | | | | | | | | |
| --- | --- | --- | --- | --- | --- | --- | --- | --- |
|  | | | | **95% Confidence Intervals** | |  | | |
| **Names** | **Effect** | **Estimate** | **SE** | **Lower** | **Upper** | **df** | **t** | **p** |
| (Intercept) | (Intercept) | 5.1048 | 0.1678 | 4.7715 | 5.4380 | 46.0 | 30.425 | <.001 |
| Age | Age | 0.1192 | 0.0473 | 0.0252 | 0.2133 | 46.0 | 2.519 | 0.015 |
| Time1 | pre - post | 6.8200 | 0.2708 | 6.2821 | 7.3579 | 47.0 | 25.184 | <.001 |
| Treatment1 | 2 - 1 | 0.2118 | 0.3356 | -0.4547 | 0.8784 | 46.0 | 0.631 | 0.531 |
| Time1 ✻ Treatment1 | (pre - post) ✻ (2 - 1) | 0.1786 | 0.5677 | -0.9490 | 1.3062 | 47.0 | 0.315 | 0.754 |
| Age ✻ Time1 | Age ✻ (pre - post) | -0.1848 | 0.0746 | -0.3329 | -0.0367 | 47.0 | -2.479 | 0.017 |
| Age ✻ Treatment1 | Age ✻ (2 - 1) | -0.0794 | 0.0947 | -0.2674 | 0.1087 | 46.0 | -0.838 | 0.406 |

| Random Components | | | | |
| --- | --- | --- | --- | --- |
| **Groups** | **Name** | **Variance** | **SD** | **ICC** |
| **Subjects** | (Intercept) | 0.345 | 0.588 | 0.158 |
| **Residual** |  | 1.833 | 1.354 |  |
| Nota. Number of Obs: 100 , Number of groups: Subjects 50 | | | | |

**Post Hoc Tests**

| Post Hoc comparison: Time ✻ Treatment | | | | | | | | | |
| --- | --- | --- | --- | --- | --- | --- | --- | --- | --- |
| **Comparison** | | | | |  | | | | |
| **Time** | **Treatment** | **vs** | **Time** | **Treatment** | **Difference** | **SE** | **t** | **df** | **p_bonferroni_** |
| post | 1 | - | post | 2 | -0.123 | 0.440 | -0.279 | 90.2 | 1.000 |
| post | 1 | - | pre | 1 | -6.731 | 0.392 | -17.157 | 47.0 | <.001 |
| post | 1 | - | pre | 2 | -7.032 | 0.431 | -16.307 | 88.6 | <.001 |
| post | 2 | - | pre | 1 | -6.608 | 0.431 | -15.325 | 88.6 | <.001 |
| post | 2 | - | pre | 2 | -6.909 | 0.392 | -17.612 | 47.0 | <.001 |
| pre | 1 | - | pre | 2 | -0.301 | 0.440 | -0.685 | 90.2 | 1.000 |

**Linear Mixed Model PCL-5**

| Model Info | | |
| --- | --- | --- |
| **Info** |  |  |
| Model Type | Mixed Model | Linear Mixed model for continuous y |
| Model | lmer | `PCL-5` ~ 1 + Age + Time + Treatment + Time:Treatment + Age:Time + Age:Treatment + ( 1 \| Subjects ) |
| Distribution | Gaussian | Normal distribution of residuals |
| Direction | y | Dependend variable scores |
| Optimizer | bobyqa |  |
| DF method | Satterthwaite |  |
| Sample size | 100 |  |
| Converged | yes |  |
| Y transform | none |  |
| C.I. method | Wald |  |
| Nota. All covariates are centered to the mean | | |

**Model Results**

| Model Fit | | | | |
| --- | --- | --- | --- | --- |
| **Type** | **R²** | **df** | **LRT X²** | **p** |
| Conditional | 0.705 | 7 | 79.147 | <.001 |
| Marginal | 0.484 | 6 | 79.147 | <.001 |

| Fixed Effects Omnibus Tests | | | | |
| --- | --- | --- | --- | --- |
|  | **F** | **df** | **df (res)** | **p** |
| **Age** | 2.36e-4 | 1 | 46.0 | 0.988 |
| **Time** | 149.42272 | 1 | 47.0 | <.001 |
| **Treatment** | 0.13374 | 1 | 46.0 | 0.716 |
| **Time ✻ Treatment** | 0.05570 | 1 | 47.0 | 0.814 |
| **Age ✻ Time** | 0.00121 | 1 | 47.0 | 0.972 |
| **Age ✻ Treatment** | 4.29352 | 1 | 46.0 | 0.044 |

| Parameter Estimates (Fixed coefficients) | | | | | | | | |
| --- | --- | --- | --- | --- | --- | --- | --- | --- |
|  | | | | **95% Confidence Intervals** | |  | | |
| **Names** | **Effect** | **Estimate** | **SE** | **Lower** | **Upper** | **df** | **t** | **p** |
| (Intercept) | (Intercept) | 19.87444 | 1.552 | 16.7910 | 22.958 | 46.0 | 12.8033 | <.001 |
| Age | Age | 0.00673 | 0.438 | -0.8631 | 0.877 | 46.0 | 0.0154 | 0.988 |
| Time1 | pre - post | 22.72000 | 1.859 | 19.0280 | 26.412 | 47.0 | 12.2239 | <.001 |
| Treatment1 | 2 - 1 | 1.13535 | 3.105 | -5.0315 | 7.302 | 46.0 | 0.3657 | 0.716 |
| Time1 ✻ Treatment1 | (pre - post) ✻ (2 - 1) | -0.91949 | 3.896 | -8.6587 | 6.820 | 47.0 | -0.2360 | 0.814 |
| Age ✻ Time1 | Age ✻ (pre - post) | 0.01777 | 0.512 | -0.9987 | 1.034 | 47.0 | 0.0347 | 0.972 |
| Age ✻ Treatment1 | Age ✻ (2 - 1) | 1.81480 | 0.876 | 0.0751 | 3.555 | 46.0 | 2.0721 | 0.044 |

| Random Components | | | | |
| --- | --- | --- | --- | --- |
| **Groups** | **Name** | **Variance** | **SD** | **ICC** |
| **Subjects** | (Intercept) | 64.8 | 8.05 | 0.429 |
| **Residual** |  | 86.4 | 9.29 |  |
| Nota. Number of Obs: 100 , Number of groups: Subjects 50 | | | | |

**Post Hoc Tests**

| Post Hoc comparison: Time ✻ Treatment | | | | | | | | | |
| --- | --- | --- | --- | --- | --- | --- | --- | --- | --- |
| **Comparison** | | | | |  | | | | |
| **Time** | **Treatment** | **vs** | **Time** | **Treatment** | **Difference** | **SE** | **t** | **df** | **p_bonferroni_** |
| post | 1 | - | post | 2 | -1.595 | 3.67 | -0.435 | 77.6 | 1.000 |
| post | 1 | - | pre | 1 | -23.180 | 2.69 | -8.609 | 47.0 | <.001 |
| post | 1 | - | pre | 2 | -23.855 | 3.62 | -6.593 | 75.4 | <.001 |
| post | 2 | - | pre | 1 | -21.585 | 3.62 | -5.965 | 75.4 | <.001 |
| post | 2 | - | pre | 2 | -22.260 | 2.69 | -8.267 | 47.0 | <.001 |
| pre | 1 | - | pre | 2 | -0.676 | 3.67 | -0.184 | 77.6 | 1.000 |

**Linear Mixed Model IES-R**

| Model Info | | |
| --- | --- | --- |
| **Info** |  |  |
| Model Type | Mixed Model | Linear Mixed model for continuous y |
| Model | lmer | `IES-R` ~ 1 + Age + Time + Treatment + Time:Treatment + Age:Time + Age:Treatment + ( 1 \| Subjects ) |
| Distribution | Gaussian | Normal distribution of residuals |
| Direction | y | Dependend variable scores |
| Optimizer | bobyqa |  |
| DF method | Satterthwaite |  |
| Sample size | 100 |  |
| Converged | yes |  |
| Y transform | none |  |
| C.I. method | Wald |  |
| Nota. All covariates are centered to the mean | | |

**Model Results**

| Model Fit | | | | |
| --- | --- | --- | --- | --- |
| **Type** | **R²** | **df** | **LRT X²** | **p** |
| Conditional | 0.737 | 7 | 97.234 | <.001 |
| Marginal | 0.577 | 6 | 97.234 | <.001 |

| Fixed Effects Omnibus Tests | | | | |
| --- | --- | --- | --- | --- |
|  | **F** | **df** | **df (res)** | **p** |
| **Age** | 0.1291 | 1 | 46.0 | 0.721 |
| **Time** | 206.4809 | 1 | 47.0 | <.001 |
| **Treatment** | 0.0533 | 1 | 46.0 | 0.818 |
| **Time ✻ Treatment** | 1.2284 | 1 | 47.0 | 0.273 |
| **Age ✻ Time** | 0.8272 | 1 | 47.0 | 0.368 |
| **Age ✻ Treatment** | 3.8266 | 1 | 46.0 | 0.057 |

| Parameter Estimates (Fixed coefficients) | | | | | | | | |
| --- | --- | --- | --- | --- | --- | --- | --- | --- |
|  | | | | **95% Confidence Intervals** | |  | | |
| **Names** | **Effect** | **Estimate** | **SE** | **Lower** | **Upper** | **df** | **t** | **p** |
| (Intercept) | (Intercept) | 25.757 | 1.616 | 22.5463 | 28.967 | 46.0 | 15.936 | <.001 |
| Age | Age | -0.164 | 0.456 | -1.0695 | 0.742 | 46.0 | -0.359 | 0.721 |
| Time1 | pre - post | 29.520 | 2.054 | 25.4393 | 33.601 | 47.0 | 14.369 | <.001 |
| Treatment1 | 2 - 1 | 0.746 | 3.232 | -5.6744 | 7.167 | 46.0 | 0.231 | 0.818 |
| Time1 ✻ Treatment1 | (pre - post) ✻ (2 - 1) | -4.773 | 4.306 | -13.3269 | 3.781 | 47.0 | -1.108 | 0.273 |
| Age ✻ Time1 | Age ✻ (pre - post) | -0.514 | 0.566 | -1.6380 | 0.609 | 47.0 | -0.910 | 0.368 |
| Age ✻ Treatment1 | Age ✻ (2 - 1) | 1.784 | 0.912 | -0.0276 | 3.595 | 46.0 | 1.956 | 0.057 |

| Random Components | | | | |
| --- | --- | --- | --- | --- |
| **Groups** | **Name** | **Variance** | **SD** | **ICC** |
| **Subjects** | (Intercept) | 64.3 | 8.02 | 0.379 |
| **Residual** |  | 105.5 | 10.27 |  |
| Nota. Number of Obs: 100 , Number of groups: Subjects 50 | | | | |

**Post Hoc Tests**

| Post Hoc comparison: Time ✻ Treatment | | | | | | | | | |
| --- | --- | --- | --- | --- | --- | --- | --- | --- | --- |
| **Comparison** | | | | |  | | | | |
| **Time** | **Treatment** | **vs** | **Time** | **Treatment** | **Difference** | **SE** | **t** | **df** | **p_bonferroni_** |
| post | 1 | - | post | 2 | -3.13 | 3.88 | -0.807 | 80.4 | 1.000 |
| post | 1 | - | pre | 1 | -31.91 | 2.98 | -10.721 | 47.0 | <.001 |
| post | 1 | - | pre | 2 | -30.27 | 3.83 | -7.902 | 78.2 | <.001 |
| post | 2 | - | pre | 1 | -28.77 | 3.83 | -7.513 | 78.2 | <.001 |
| post | 2 | - | pre | 2 | -27.13 | 2.98 | -9.117 | 47.0 | <.001 |
| pre | 1 | - | pre | 2 | 1.64 | 3.88 | 0.422 | 80.4 | 1.000 |

**Linear Mixed Model IES-R (Avoidance)**

| Model Info | | |
| --- | --- | --- |
| **Info** |  |  |
| Model Type | Mixed Model | Linear Mixed model for continuous y |
| Model | lmer | `IES-R (Ev)` ~ 1 + Age + Time + Treatment + Time:Treatment + Age:Time + Age:Treatment + ( 1 \| Subjects ) |
| Distribution | Gaussian | Normal distribution of residuals |
| Direction | y | Dependend variable scores |
| Optimizer | bobyqa |  |
| DF method | Satterthwaite |  |
| Sample size | 100 |  |
| Converged | yes |  |
| Y transform | none |  |
| C.I. method | Wald |  |
| Nota. All covariates are centered to the mean | | |

**Model Results**

| Model Fit | | | | |
| --- | --- | --- | --- | --- |
| **Type** | **R²** | **df** | **LRT X²** | **p** |
| Conditional | 0.702 | 7 | 82.333 | <.001 |
| Marginal | 0.507 | 6 | 82.333 | <.001 |

| Fixed Effects Omnibus Tests | | | | |
| --- | --- | --- | --- | --- |
|  | **F** | **df** | **df (res)** | **p** |
| **Age** | 0.0535 | 1 | 46.0 | 0.818 |
| **Time** | 159.4164 | 1 | 47.0 | <.001 |
| **Treatment** | 0.3743 | 1 | 46.0 | 0.544 |
| **Time ✻ Treatment** | 1.3917 | 1 | 47.0 | 0.244 |
| **Age ✻ Time** | 1.8346 | 1 | 47.0 | 0.182 |
| **Age ✻ Treatment** | 1.9292 | 1 | 46.0 | 0.172 |

| Parameter Estimates (Fixed coefficients) | | | | | | | | |
| --- | --- | --- | --- | --- | --- | --- | --- | --- |
|  | | | | **95% Confidence Intervals** | |  | | |
| **Names** | **Effect** | **Estimate** | **SE** | **Lower** | **Upper** | **df** | **t** | **p** |
| (Intercept) | (Intercept) | 1.19033 | 0.0791 | 1.0332 | 1.3474 | 46.0 | 15.051 | <.001 |
| Age | Age | 0.00516 | 0.0223 | -0.0392 | 0.0495 | 46.0 | 0.231 | 0.818 |
| Time1 | pre - post | 1.24500 | 0.0986 | 1.0491 | 1.4409 | 47.0 | 12.626 | <.001 |
| Treatment1 | 2 - 1 | 0.09677 | 0.1582 | -0.2174 | 0.4110 | 46.0 | 0.612 | 0.544 |
| Time1 ✻ Treatment1 | (pre - post) ✻ (2 - 1) | -0.24384 | 0.2067 | -0.6544 | 0.1667 | 47.0 | -1.180 | 0.244 |
| Age ✻ Time1 | Age ✻ (pre - post) | -0.03677 | 0.0271 | -0.0907 | 0.0172 | 47.0 | -1.354 | 0.182 |
| Age ✻ Treatment1 | Age ✻ (2 - 1) | 0.06198 | 0.0446 | -0.0267 | 0.1506 | 46.0 | 1.389 | 0.172 |

| Random Components | | | | |
| --- | --- | --- | --- | --- |
| **Groups** | **Name** | **Variance** | **SD** | **ICC** |
| **Subjects** | (Intercept) | 0.159 | 0.399 | 0.395 |
| **Residual** |  | 0.243 | 0.493 |  |
| Nota. Number of Obs: 100 , Number of groups: Subjects 50 | | | | |

**Post Hoc Tests**

| Post Hoc comparison: Time ✻ Treatment | | | | | | | | | |
| --- | --- | --- | --- | --- | --- | --- | --- | --- | --- |
| **Comparison** | | | | |  | | | | |
| **Time** | **Treatment** | **vs** | **Time** | **Treatment** | **Difference** | **SE** | **t** | **df** | **p_bonferroni_** |
| post | 1 | - | post | 2 | -0.2187 | 0.189 | -1.157 | 79.5 | 1.000 |
| post | 1 | - | pre | 1 | -1.3669 | 0.143 | -9.569 | 47.0 | <.001 |
| post | 1 | - | pre | 2 | -1.3418 | 0.186 | -7.199 | 77.3 | <.001 |
| post | 2 | - | pre | 1 | -1.1482 | 0.186 | -6.160 | 77.3 | <.001 |
| post | 2 | - | pre | 2 | -1.1231 | 0.143 | -7.862 | 47.0 | <.001 |
| pre | 1 | - | pre | 2 | 0.0251 | 0.189 | 0.133 | 79.5 | 1.000 |

**Linear Mixed Model IES-R (Re-experiencing)**

| Model Info | | |
| --- | --- | --- |
| **Info** |  |  |
| Model Type | Mixed Model | Linear Mixed model for continuous y |
| Model | lmer | `IES-R (In)` ~ 1 + Age + Time + Treatment + Time:Treatment + Age:Time + Age:Treatment + ( 1 \| Subjects ) |
| Distribution | Gaussian | Normal distribution of residuals |
| Direction | y | Dependend variable scores |
| Optimizer | bobyqa |  |
| DF method | Satterthwaite |  |
| Sample size | 100 |  |
| Converged | yes |  |
| Y transform | none |  |
| C.I. method | Wald |  |
| Nota. All covariates are centered to the mean | | |

**Model Results**

| Model Fit | | | | |
| --- | --- | --- | --- | --- |
| **Type** | **R²** | **df** | **LRT X²** | **p** |
| Conditional | 0.691 | 7 | 94.209 | <.001 |
| Marginal | 0.581 | 6 | 94.209 | <.001 |

| Fixed Effects Omnibus Tests | | | | |
| --- | --- | --- | --- | --- |
|  | **F** | **df** | **df (res)** | **p** |
| **Age** | 0.0535 | 1 | 46.0 | 0.818 |
| **Time** | 176.6813 | 1 | 47.0 | <.001 |
| **Treatment** | 0.0918 | 1 | 46.0 | 0.763 |
| **Time ✻ Treatment** | 0.6067 | 1 | 47.0 | 0.440 |
| **Age ✻ Time** | 0.1329 | 1 | 47.0 | 0.717 |
| **Age ✻ Treatment** | 4.6546 | 1 | 46.0 | 0.036 |

| Parameter Estimates (Fixed coefficients) | | | | | | | | |
| --- | --- | --- | --- | --- | --- | --- | --- | --- |
|  | | | | **95% Confidence Intervals** | |  | | |
| **Names** | **Effect** | **Estimate** | **SE** | **Lower** | **Upper** | **df** | **t** | **p** |
| (Intercept) | (Intercept) | 1.24412 | 0.0762 | 1.09277 | 1.3955 | 46.0 | 16.328 | <.001 |
| Age | Age | -0.00497 | 0.0215 | -0.04767 | 0.0377 | 46.0 | -0.231 | 0.818 |
| Time1 | pre - post | 1.46750 | 0.1104 | 1.24820 | 1.6868 | 47.0 | 13.292 | <.001 |
| Treatment1 | 2 - 1 | 0.04616 | 0.1524 | -0.25654 | 0.3489 | 46.0 | 0.303 | 0.763 |
| Time1 ✻ Treatment1 | (pre - post) ✻ (2 - 1) | -0.18027 | 0.2314 | -0.63997 | 0.2794 | 47.0 | -0.779 | 0.440 |
| Age ✻ Time1 | Age ✻ (pre - post) | -0.01108 | 0.0304 | -0.07146 | 0.0493 | 47.0 | -0.365 | 0.717 |
| Age ✻ Treatment1 | Age ✻ (2 - 1) | 0.09275 | 0.0430 | 0.00735 | 0.1781 | 46.0 | 2.157 | 0.036 |

| Random Components | | | | |
| --- | --- | --- | --- | --- |
| **Groups** | **Name** | **Variance** | **SD** | **ICC** |
| **Subjects** | (Intercept) | 0.108 | 0.328 | 0.261 |
| **Residual** |  | 0.305 | 0.552 |  |
| Nota. Number of Obs: 100 , Number of groups: Subjects 50 | | | | |

**Post Hoc Tests**

| Post Hoc comparison: Time ✻ Treatment | | | | | | | | | |
| --- | --- | --- | --- | --- | --- | --- | --- | --- | --- |
| **Comparison** | | | | |  | | | | |
| **Time** | **Treatment** | **vs** | **Time** | **Treatment** | **Difference** | **SE** | **t** | **df** | **p_bonferroni_** |
| post | 1 | - | post | 2 | -0.1363 | 0.191 | -0.712 | 86.3 | 1.000 |
| post | 1 | - | pre | 1 | -1.5576 | 0.160 | -9.739 | 47.0 | <.001 |
| post | 1 | - | pre | 2 | -1.5137 | 0.188 | -8.044 | 84.2 | <.001 |
| post | 2 | - | pre | 1 | -1.4213 | 0.188 | -7.553 | 84.2 | <.001 |
| post | 2 | - | pre | 2 | -1.3774 | 0.160 | -8.612 | 47.0 | <.001 |
| pre | 1 | - | pre | 2 | 0.0440 | 0.191 | 0.230 | 86.3 | 1.000 |

**Linear Mixed Model IES-R (Hyperarousal)**

| Model Info | | |
| --- | --- | --- |
| **Info** |  |  |
| Model Type | Mixed Model | Linear Mixed model for continuous y |
| Model | lmer | `IES-R (IpA)` ~ 1 + Age + Time + Treatment + Time:Treatment + Age:Time + Age:Treatment + ( 1 \| Subjects ) |
| Distribution | Gaussian | Normal distribution of residuals |
| Direction | y | Dependend variable scores |
| Optimizer | bobyqa |  |
| DF method | Satterthwaite |  |
| Sample size | 100 |  |
| Converged | yes |  |
| Y transform | none |  |
| C.I. method | Wald |  |
| Nota. All covariates are centered to the mean | | |

**Model Results**

| Model Fit | | | | |
| --- | --- | --- | --- | --- |
| **Type** | **R²** | **df** | **LRT X²** | **p** |
| Conditional | 0.685 | 7 | 75.957 | <.001 |
| Marginal | 0.475 | 6 | 75.957 | <.001 |

| Fixed Effects Omnibus Tests | | | | |
| --- | --- | --- | --- | --- |
|  | **F** | **df** | **df (res)** | **p** |
| **Age** | 1.210 | 1 | 46.0 | 0.277 |
| **Time** | 139.879 | 1 | 47.0 | <.001 |
| **Treatment** | 0.139 | 1 | 46.0 | 0.711 |
| **Time ✻ Treatment** | 0.991 | 1 | 47.0 | 0.324 |
| **Age ✻ Time** | 0.523 | 1 | 47.0 | 0.473 |
| **Age ✻ Treatment** | 3.300 | 1 | 46.0 | 0.076 |

| Parameter Estimates (Fixed coefficients) | | | | | | | | |
| --- | --- | --- | --- | --- | --- | --- | --- | --- |
|  | | | | **95% Confidence Intervals** | |  | | |
| **Names** | **Effect** | **Estimate** | **SE** | **Lower** | **Upper** | **df** | **t** | **p** |
| (Intercept) | (Intercept) | 1.0469 | 0.0888 | 0.87050 | 1.2232 | 46.0 | 11.790 | <.001 |
| Age | Age | -0.0276 | 0.0250 | -0.07731 | 0.0222 | 46.0 | -1.100 | 0.277 |
| Time1 | pre - post | 1.3033 | 0.1102 | 1.08444 | 1.5222 | 47.0 | 11.827 | <.001 |
| Treatment1 | 2 - 1 | -0.0662 | 0.1776 | -0.41891 | 0.2866 | 46.0 | -0.373 | 0.711 |
| Time1 ✻ Treatment1 | (pre - post) ✻ (2 - 1) | -0.2300 | 0.2310 | -0.68886 | 0.2288 | 47.0 | -0.996 | 0.324 |
| Age ✻ Time1 | Age ✻ (pre - post) | -0.0219 | 0.0303 | -0.08220 | 0.0383 | 47.0 | -0.723 | 0.473 |
| Age ✻ Treatment1 | Age ✻ (2 - 1) | 0.0910 | 0.0501 | -0.00851 | 0.1905 | 46.0 | 1.817 | 0.076 |

| Random Components | | | | |
| --- | --- | --- | --- | --- |
| **Groups** | **Name** | **Variance** | **SD** | **ICC** |
| **Subjects** | (Intercept) | 0.202 | 0.449 | 0.399 |
| **Residual** |  | 0.304 | 0.551 |  |
| Nota. Number of Obs: 100 , Number of groups: Subjects 50 | | | | |

**Post Hoc Tests**

| Post Hoc comparison: Time ✻ Treatment | | | | | | | | | |
| --- | --- | --- | --- | --- | --- | --- | --- | --- | --- |
| **Comparison** | | | | |  | | | | |
| **Time** | **Treatment** | **vs** | **Time** | **Treatment** | **Difference** | **SE** | **t** | **df** | **p_bonferroni_** |
| post | 1 | - | post | 2 | -0.0488 | 0.212 | -0.231 | 79.3 | 1.000 |
| post | 1 | - | pre | 1 | -1.4183 | 0.160 | -8.885 | 47.0 | <.001 |
| post | 1 | - | pre | 2 | -1.2372 | 0.209 | -5.920 | 77.1 | <.001 |
| post | 2 | - | pre | 1 | -1.3695 | 0.209 | -6.553 | 77.1 | <.001 |
| post | 2 | - | pre | 2 | -1.1883 | 0.160 | -7.444 | 47.0 | <.001 |
| pre | 1 | - | pre | 2 | 0.1812 | 0.212 | 0.855 | 79.3 | 1.000 |

**Linear Mixed Model EMDR-Posner Sex**

**Linear Mixed Model SUDs**

| Model Info | | |
| --- | --- | --- |
| **Info** |  |  |
| Model Type | Mixed Model | Linear Mixed model for continuous y |
| Model | lmer | SUDs ~ 1 + Time + Treatment + Sex + Time:Treatment + Time:Sex + Treatment:Sex + Time:Treatment:Sex + ( 1 \| Subjects ) |
| Distribution | Gaussian | Normal distribution of residuals |
| Direction | y | Dependend variable scores |
| Optimizer | bobyqa |  |
| DF method | Satterthwaite |  |
| Sample size | 100 |  |
| Converged | yes |  |
| Y transform | none |  |
| C.I. method | Wald |  |

**Model Results**

| Model Fit | | | | |
| --- | --- | --- | --- | --- |
| **Type** | **R²** | **df** | **LRT X²** | **p** |
| Conditional | 0.860 | 8 | 185.270 | <.001 |
| Marginal | 0.831 | 7 | 185.270 | <.001 |

| Fixed Effects Omnibus Tests | | | | |
| --- | --- | --- | --- | --- |
|  | **F** | **df** | **df (res)** | **p** |
| **Time** | 578.4989 | 1 | 46.0 | <.001 |
| **Treatment** | 0.0254 | 1 | 46.0 | 0.874 |
| **Sex** | 0.5585 | 1 | 46.0 | 0.459 |
| **Time ✻ Treatment** | 1.3277 | 1 | 46.0 | 0.255 |
| **Time ✻ Sex** | 2.7166 | 1 | 46.0 | 0.106 |
| **Treatment ✻ Sex** | 0.1262 | 1 | 46.0 | 0.724 |
| **Time ✻ Treatment ✻ Sex** | 0.0615 | 1 | 46.0 | 0.805 |

| Parameter Estimates (Fixed coefficients) | | | | | | | | |
| --- | --- | --- | --- | --- | --- | --- | --- | --- |
|  | | | | **95% Confidence Intervals** | |  | | |
| **Names** | **Effect** | **Estimate** | **SE** | **Lower** | **Upper** | **df** | **t** | **p** |
| (Intercept) | (Intercept) | 5.1604 | 0.170 | 4.823 | 5.498 | 46.0 | 30.348 | <.001 |
| Time1 | pre - post | 6.8708 | 0.286 | 6.303 | 7.438 | 46.0 | 24.052 | <.001 |
| Treatment1 | 2 - 1 | -0.0542 | 0.340 | -0.730 | 0.621 | 46.0 | -0.159 | 0.874 |
| Sex1 | 1 - 0 | 0.2542 | 0.340 | -0.421 | 0.930 | 46.0 | 0.747 | 0.459 |
| Time1 ✻ Treatment1 | (pre - post) ✻ (2 - 1) | 0.6583 | 0.571 | -0.477 | 1.793 | 46.0 | 1.152 | 0.255 |
| Time1 ✻ Sex1 | (pre - post) ✻ (1 - 0) | 0.9417 | 0.571 | -0.193 | 2.077 | 46.0 | 1.648 | 0.106 |
| Treatment1 ✻ Sex1 | (2 - 1) ✻ (1 - 0) | -0.2417 | 0.680 | -1.593 | 1.110 | 46.0 | -0.355 | 0.724 |
| Time1 ✻ Treatment1 ✻ Sex1 | (pre - post) ✻ (2 - 1) ✻ (1 - 0) | -0.2833 | 1.143 | -2.553 | 1.987 | 46.0 | -0.248 | 0.805 |

| Random Components | | | | |
| --- | --- | --- | --- | --- |
| **Groups** | **Name** | **Variance** | **SD** | **ICC** |
| **Subjects** | (Intercept) | 0.417 | 0.645 | 0.173 |
| **Residual** |  | 1.997 | 1.413 |  |
| Nota. Number of Obs: 100 , Number of groups: Subjects 50 | | | | |

**Post Hoc Tests**

| Post Hoc comparison: Time ✻ Treatment | | | | | | | | | |
| --- | --- | --- | --- | --- | --- | --- | --- | --- | --- |
| **Comparison** | | | | |  | | | | |
| **Time** | **Treatment** | **vs** | **Time** | **Treatment** | **Difference** | **SE** | **t** | **df** | **p_bonferroni_** |
| post | 1 | - | post | 2 | 0.383 | 0.444 | 0.863 | 89.3 | 1.000 |
| post | 1 | - | pre | 1 | -6.542 | 0.400 | -16.354 | 46.0 | <.001 |
| post | 1 | - | pre | 2 | -6.817 | 0.444 | -15.348 | 89.3 | <.001 |
| post | 2 | - | pre | 1 | -6.925 | 0.444 | -15.592 | 89.3 | <.001 |
| post | 2 | - | pre | 2 | -7.200 | 0.408 | -17.650 | 46.0 | <.001 |
| pre | 1 | - | pre | 2 | -0.275 | 0.444 | -0.619 | 89.3 | 1.000 |

**Linear Mixed Model PCL-5**

| Model Info | | |
| --- | --- | --- |
| **Info** |  |  |
| Model Type | Mixed Model | Linear Mixed model for continuous y |
| Model | lmer | `PCL-5` ~ 1 + Time + Treatment + Sex + Time:Treatment + Time:Sex + Treatment:Sex + Time:Treatment:Sex + ( 1 \| Subjects ) |
| Distribution | Gaussian | Normal distribution of residuals |
| Direction | y | Dependend variable scores |
| Optimizer | bobyqa |  |
| DF method | Satterthwaite |  |
| Sample size | 100 |  |
| Converged | yes |  |
| Y transform | none |  |
| C.I. method | Wald |  |

**Model Results**

| Model Fit | | | | |
| --- | --- | --- | --- | --- |
| **Type** | **R²** | **df** | **LRT X²** | **p** |
| Conditional | 0.714 | 8 | 78.062 | <.001 |
| Marginal | 0.464 | 7 | 78.062 | <.001 |

| Fixed Effects Omnibus Tests | | | | |
| --- | --- | --- | --- | --- |
|  | **F** | **df** | **df (res)** | **p** |
| **Time** | 154.86506 | 1 | 46.0 | <.001 |
| **Treatment** | 0.30272 | 1 | 46.0 | 0.585 |
| **Sex** | 0.23011 | 1 | 46.0 | 0.634 |
| **Time ✻ Treatment** | 0.00935 | 1 | 46.0 | 0.923 |
| **Time ✻ Sex** | 2.01908 | 1 | 46.0 | 0.162 |
| **Treatment ✻ Sex** | 1.50910 | 1 | 46.0 | 0.226 |
| **Time ✻ Treatment ✻ Sex** | 0.16163 | 1 | 46.0 | 0.690 |

| Parameter Estimates (Fixed coefficients) | | | | | | | | |
| --- | --- | --- | --- | --- | --- | --- | --- | --- |
|  | | | | **95% Confidence Intervals** | |  | | |
| **Names** | **Effect** | **Estimate** | **SE** | **Lower** | **Upper** | **df** | **t** | **p** |
| (Intercept) | (Intercept) | 19.079 | 1.54 | 16.03 | 22.13 | 46.0 | 12.4155 | <.001 |
| Time1 | pre - post | 23.096 | 1.86 | 19.41 | 26.78 | 46.0 | 12.4445 | <.001 |
| Treatment1 | 2 - 1 | 1.691 | 3.07 | -4.41 | 7.80 | 46.0 | 0.5502 | 0.585 |
| Sex1 | 1 - 0 | 1.474 | 3.07 | -4.63 | 7.58 | 46.0 | 0.4797 | 0.634 |
| Time1 ✻ Treatment1 | (pre - post) ✻ (2 - 1) | -0.359 | 3.71 | -7.73 | 7.02 | 46.0 | -0.0967 | 0.923 |
| Time1 ✻ Sex1 | (pre - post) ✻ (1 - 0) | 5.274 | 3.71 | -2.10 | 12.65 | 46.0 | 1.4209 | 0.162 |
| Treatment1 ✻ Sex1 | (2 - 1) ✻ (1 - 0) | 7.551 | 6.15 | -4.66 | 19.76 | 46.0 | 1.2285 | 0.226 |
| Time1 ✻ Treatment1 ✻ Sex1 | (pre - post) ✻ (2 - 1) ✻ (1 - 0) | 2.985 | 7.42 | -11.76 | 17.73 | 46.0 | 0.4020 | 0.690 |

| Random Components | | | | |
| --- | --- | --- | --- | --- |
| **Groups** | **Name** | **Variance** | **SD** | **ICC** |
| **Subjects** | (Intercept) | 73.4 | 8.57 | 0.466 |
| **Residual** |  | 84.3 | 9.18 |  |
| Nota. Number of Obs: 100 , Number of groups: Subjects 50 | | | | |

**Post Hoc Tests**

| Post Hoc comparison: Time ✻ Treatment | | | | | | | | | |
| --- | --- | --- | --- | --- | --- | --- | --- | --- | --- |
| **Comparison** | | | | |  | | | | |
| **Time** | **Treatment** | **vs** | **Time** | **Treatment** | **Difference** | **SE** | **t** | **df** | **p_bonferroni_** |
| post | 1 | - | post | 2 | -1.87 | 3.59 | -0.521 | 75.6 | 1.000 |
| post | 1 | - | pre | 1 | -23.28 | 2.60 | -8.956 | 46.0 | <.001 |
| post | 1 | - | pre | 2 | -24.79 | 3.59 | -6.904 | 75.6 | <.001 |
| post | 2 | - | pre | 1 | -21.41 | 3.59 | -5.962 | 75.6 | <.001 |
| post | 2 | - | pre | 2 | -22.92 | 2.65 | -8.647 | 46.0 | <.001 |
| pre | 1 | - | pre | 2 | -1.51 | 3.59 | -0.421 | 75.6 | 1.000 |

**Linear Mixed Model IES-R**

| Model Info | | |
| --- | --- | --- |
| **Info** |  |  |
| Model Type | Mixed Model | Linear Mixed model for continuous y |
| Model | lmer | `IES-R` ~ 1 + Time + Treatment + Sex + Time:Treatment + Time:Sex + Treatment:Sex + Time:Treatment:Sex + ( 1 \| Subjects ) |
| Distribution | Gaussian | Normal distribution of residuals |
| Direction | y | Dependend variable scores |
| Optimizer | bobyqa |  |
| DF method | Satterthwaite |  |
| Sample size | 100 |  |
| Converged | yes |  |
| Y transform | none |  |
| C.I. method | Wald |  |

**Model Results**

| Model Fit | | | | |
| --- | --- | --- | --- | --- |
| **Type** | **R²** | **df** | **LRT X²** | **p** |
| Conditional | 0.733 | 8 | 95.096 | <.001 |
| Marginal | 0.563 | 7 | 95.096 | <.001 |

| Fixed Effects Omnibus Tests | | | | |
| --- | --- | --- | --- | --- |
|  | **F** | **df** | **df (res)** | **p** |
| **Time** | 200.9491 | 1 | 46.0 | <.001 |
| **Treatment** | 0.2278 | 1 | 46.0 | 0.635 |
| **Sex** | 0.1538 | 1 | 46.0 | 0.697 |
| **Time ✻ Treatment** | 0.5331 | 1 | 46.0 | 0.469 |
| **Time ✻ Sex** | 0.0535 | 1 | 46.0 | 0.818 |
| **Treatment ✻ Sex** | 1.6587 | 1 | 46.0 | 0.204 |
| **Time ✻ Treatment ✻ Sex** | 0.8334 | 1 | 46.0 | 0.366 |

| Parameter Estimates (Fixed coefficients) | | | | | | | | |
| --- | --- | --- | --- | --- | --- | --- | --- | --- |
|  | | | | **95% Confidence Intervals** | |  | | |
| **Names** | **Effect** | **Estimate** | **SE** | **Lower** | **Upper** | **df** | **t** | **p** |
| (Intercept) | (Intercept) | 24.829 | 1.58 | 21.69 | 27.97 | 46.0 | 15.699 | <.001 |
| Time1 | pre - post | 29.731 | 2.10 | 25.56 | 33.90 | 46.0 | 14.176 | <.001 |
| Treatment1 | 2 - 1 | 1.510 | 3.16 | -4.77 | 7.79 | 46.0 | 0.477 | 0.635 |
| Sex1 | 1 - 0 | -1.240 | 3.16 | -7.52 | 5.04 | 46.0 | -0.392 | 0.697 |
| Time1 ✻ Treatment1 | (pre - post) ✻ (2 - 1) | -3.063 | 4.19 | -11.40 | 5.27 | 46.0 | -0.730 | 0.469 |
| Time1 ✻ Sex1 | (pre - post) ✻ (1 - 0) | 0.971 | 4.19 | -7.36 | 9.30 | 46.0 | 0.231 | 0.818 |
| Treatment1 ✻ Sex1 | (2 - 1) ✻ (1 - 0) | 8.147 | 6.33 | -4.42 | 20.72 | 46.0 | 1.288 | 0.204 |
| Time1 ✻ Treatment1 ✻ Sex1 | (pre - post) ✻ (2 - 1) ✻ (1 - 0) | 7.659 | 8.39 | -9.01 | 24.33 | 46.0 | 0.913 | 0.366 |

| Random Components | | | | |
| --- | --- | --- | --- | --- |
| **Groups** | **Name** | **Variance** | **SD** | **ICC** |
| **Subjects** | (Intercept) | 68.6 | 8.28 | 0.389 |
| **Residual** |  | 107.6 | 10.38 |  |
| Nota. Number of Obs: 100 , Number of groups: Subjects 50 | | | | |

**Post Hoc Tests**

| Post Hoc comparison: Time ✻ Treatment | | | | | | | | | |
| --- | --- | --- | --- | --- | --- | --- | --- | --- | --- |
| **Comparison** | | | | |  | | | | |
| **Time** | **Treatment** | **vs** | **Time** | **Treatment** | **Difference** | **SE** | **t** | **df** | **p_bonferroni_** |
| post | 1 | - | post | 2 | -3.0410 | 3.80 | -0.80127 | 79.9 | 1.000 |
| post | 1 | - | pre | 1 | -31.2628 | 2.94 | -10.64488 | 46.0 | <.001 |
| post | 1 | - | pre | 2 | -31.2410 | 3.80 | -8.23161 | 79.9 | <.001 |
| post | 2 | - | pre | 1 | -28.2218 | 3.80 | -7.43608 | 79.9 | <.001 |
| post | 2 | - | pre | 2 | -28.2000 | 3.00 | -9.41554 | 46.0 | <.001 |
| pre | 1 | - | pre | 2 | 0.0218 | 3.80 | 0.00574 | 79.9 | 1.000 |

**Linear Mixed Model IES-R (Avoidance)**

| Model Info | | |
| --- | --- | --- |
| **Info** |  |  |
| Model Type | Mixed Model | Linear Mixed model for continuous y |
| Model | lmer | `IES-R (Ev)` ~ 1 + Time + Treatment + Sex + Time:Treatment + Time:Sex + Treatment:Sex + Time:Treatment:Sex + ( 1 \| Subjects ) |
| Distribution | Gaussian | Normal distribution of residuals |
| Direction | y | Dependend variable scores |
| Optimizer | bobyqa |  |
| DF method | Satterthwaite |  |
| Sample size | 100 |  |
| Converged | yes |  |
| Y transform | none |  |
| C.I. method | Wald |  |

**Model Results**

| Model Fit | | | | |
| --- | --- | --- | --- | --- |
| **Type** | **R²** | **df** | **LRT X²** | **p** |
| Conditional | 0.689 | 8 | 79.475 | <.001 |
| Marginal | 0.492 | 7 | 79.475 | <.001 |

| Fixed Effects Omnibus Tests | | | | |
| --- | --- | --- | --- | --- |
|  | **F** | **df** | **df (res)** | **p** |
| **Time** | 151.4102 | 1 | 46.0 | <.001 |
| **Treatment** | 0.3188 | 1 | 46.0 | 0.575 |
| **Sex** | 0.6792 | 1 | 46.0 | 0.414 |
| **Time ✻ Treatment** | 0.4778 | 1 | 46.0 | 0.493 |
| **Time ✻ Sex** | 0.4713 | 1 | 46.0 | 0.496 |
| **Treatment ✻ Sex** | 0.4199 | 1 | 46.0 | 0.520 |
| **Time ✻ Treatment ✻ Sex** | 0.0984 | 1 | 46.0 | 0.755 |

| Parameter Estimates (Fixed coefficients) | | | | | | | | |
| --- | --- | --- | --- | --- | --- | --- | --- | --- |
|  | | | | **95% Confidence Intervals** | |  | | |
| **Names** | **Effect** | **Estimate** | **SE** | **Lower** | **Upper** | **df** | **t** | **p** |
| (Intercept) | (Intercept) | 1.1514 | 0.0769 | 0.999 | 1.304 | 46.0 | 14.976 | <.001 |
| Time1 | pre - post | 1.2560 | 0.1021 | 1.053 | 1.459 | 46.0 | 12.305 | <.001 |
| Treatment1 | 2 - 1 | 0.0868 | 0.1538 | -0.219 | 0.392 | 46.0 | 0.565 | 0.575 |
| Sex1 | 1 - 0 | -0.1267 | 0.1538 | -0.432 | 0.179 | 46.0 | -0.824 | 0.414 |
| Time1 ✻ Treatment1 | (pre - post) ✻ (2 - 1) | -0.1411 | 0.2041 | -0.547 | 0.264 | 46.0 | -0.691 | 0.493 |
| Time1 ✻ Sex1 | (pre - post) ✻ (1 - 0) | 0.1401 | 0.2041 | -0.265 | 0.546 | 46.0 | 0.687 | 0.496 |
| Treatment1 ✻ Sex1 | (2 - 1) ✻ (1 - 0) | 0.1993 | 0.3075 | -0.412 | 0.810 | 46.0 | 0.648 | 0.520 |
| Time1 ✻ Treatment1 ✻ Sex1 | (pre - post) ✻ (2 - 1) ✻ (1 - 0) | 0.1280 | 0.4083 | -0.683 | 0.939 | 46.0 | 0.314 | 0.755 |

| Random Components | | | | |
| --- | --- | --- | --- | --- |
| **Groups** | **Name** | **Variance** | **SD** | **ICC** |
| **Subjects** | (Intercept) | 0.162 | 0.402 | 0.388 |
| **Residual** |  | 0.255 | 0.505 |  |
| Nota. Number of Obs: 100 , Number of groups: Subjects 50 | | | | |

**Post Hoc Tests**

| Post Hoc comparison: Time ✻ Treatment | | | | | | | | | |
| --- | --- | --- | --- | --- | --- | --- | --- | --- | --- |
| **Comparison** | | | | |  | | | | |
| **Time** | **Treatment** | **vs** | **Time** | **Treatment** | **Difference** | **SE** | **t** | **df** | **p_bonferroni_** |
| post | 1 | - | post | 2 | -0.1574 | 0.185 | -0.8527 | 79.9 | 1.000 |
| post | 1 | - | pre | 1 | -1.3265 | 0.143 | -9.2811 | 46.0 | <.001 |
| post | 1 | - | pre | 2 | -1.3428 | 0.185 | -7.2758 | 79.9 | <.001 |
| post | 2 | - | pre | 1 | -1.1692 | 0.185 | -6.3350 | 79.9 | <.001 |
| post | 2 | - | pre | 2 | -1.1854 | 0.146 | -8.1328 | 46.0 | <.001 |
| pre | 1 | - | pre | 2 | -0.0163 | 0.185 | -0.0881 | 79.9 | 1.000 |

**Linear Mixed Model IES-R (Re-experiencing)**

| Model Info | | |
| --- | --- | --- |
| **Info** |  |  |
| Model Type | Mixed Model | Linear Mixed model for continuous y |
| Model | lmer | `IES-R (In)` ~ 1 + Time + Treatment + Sex + Time:Treatment + Time:Sex + Treatment:Sex + Time:Treatment:Sex + ( 1 \| Subjects ) |
| Distribution | Gaussian | Normal distribution of residuals |
| Direction | y | Dependend variable scores |
| Optimizer | bobyqa |  |
| DF method | Satterthwaite |  |
| Sample size | 100 |  |
| Converged | yes |  |
| Y transform | none |  |
| C.I. method | Wald |  |

**Model Results**

| Model Fit | | | | |
| --- | --- | --- | --- | --- |
| **Type** | **R²** | **df** | **LRT X²** | **p** |
| Conditional | 0.690 | 8 | 91.862 | <.001 |
| Marginal | 0.566 | 7 | 91.862 | <.001 |

| Fixed Effects Omnibus Tests | | | | |
| --- | --- | --- | --- | --- |
|  | **F** | **df** | **df (res)** | **p** |
| **Time** | 173.24457 | 1 | 46.0 | <.001 |
| **Treatment** | 0.25747 | 1 | 46.0 | 0.614 |
| **Sex** | 0.21404 | 1 | 46.0 | 0.646 |
| **Time ✻ Treatment** | 0.34986 | 1 | 46.0 | 0.557 |
| **Time ✻ Sex** | 0.00273 | 1 | 46.0 | 0.959 |
| **Treatment ✻ Sex** | 1.82128 | 1 | 46.0 | 0.184 |
| **Time ✻ Treatment ✻ Sex** | 0.75764 | 1 | 46.0 | 0.389 |

| Parameter Estimates (Fixed coefficients) | | | | | | | | |
| --- | --- | --- | --- | --- | --- | --- | --- | --- |
|  | | | | **95% Confidence Intervals** | |  | | |
| **Names** | **Effect** | **Estimate** | **SE** | **Lower** | **Upper** | **df** | **t** | **p** |
| (Intercept) | (Intercept) | 1.1952 | 0.0752 | 1.046 | 1.345 | 46.0 | 15.9004 | <.001 |
| Time1 | pre - post | 1.4746 | 0.1120 | 1.252 | 1.697 | 46.0 | 13.1622 | <.001 |
| Treatment1 | 2 - 1 | 0.0763 | 0.1503 | -0.222 | 0.375 | 46.0 | 0.5074 | 0.614 |
| Sex1 | 1 - 0 | -0.0696 | 0.1503 | -0.368 | 0.229 | 46.0 | -0.4626 | 0.646 |
| Time1 ✻ Treatment1 | (pre - post) ✻ (2 - 1) | -0.1325 | 0.2241 | -0.578 | 0.313 | 46.0 | -0.5915 | 0.557 |
| Time1 ✻ Sex1 | (pre - post) ✻ (1 - 0) | -0.0117 | 0.2241 | -0.457 | 0.433 | 46.0 | -0.0522 | 0.959 |
| Treatment1 ✻ Sex1 | (2 - 1) ✻ (1 - 0) | 0.4058 | 0.3007 | -0.192 | 1.003 | 46.0 | 1.3495 | 0.184 |
| Time1 ✻ Treatment1 ✻ Sex1 | (pre - post) ✻ (2 - 1) ✻ (1 - 0) | 0.3901 | 0.4481 | -0.500 | 1.280 | 46.0 | 0.8704 | 0.389 |

| Random Components | | | | |
| --- | --- | --- | --- | --- |
| **Groups** | **Name** | **Variance** | **SD** | **ICC** |
| **Subjects** | (Intercept) | 0.123 | 0.351 | 0.286 |
| **Residual** |  | 0.307 | 0.554 |  |
| Nota. Number of Obs: 100 , Number of groups: Subjects 50 | | | | |

**Post Hoc Tests**

| Post Hoc comparison: Time ✻ Treatment | | | | | | | | | |
| --- | --- | --- | --- | --- | --- | --- | --- | --- | --- |
| **Comparison** | | | | |  | | | | |
| **Time** | **Treatment** | **vs** | **Time** | **Treatment** | **Difference** | **SE** | **t** | **df** | **p_bonferroni_** |
| post | 1 | - | post | 2 | -0.1425 | 0.187 | -0.7603 | 85.0 | 1.000 |
| post | 1 | - | pre | 1 | -1.5409 | 0.157 | -9.8221 | 46.0 | <.001 |
| post | 1 | - | pre | 2 | -1.5509 | 0.187 | -8.2719 | 85.0 | <.001 |
| post | 2 | - | pre | 1 | -1.3983 | 0.187 | -7.4581 | 85.0 | <.001 |
| post | 2 | - | pre | 2 | -1.4083 | 0.160 | -8.8030 | 46.0 | <.001 |
| pre | 1 | - | pre | 2 | -0.0100 | 0.187 | -0.0534 | 85.0 | 1.000 |

**Linear Mixed Model IES-R (Hyperarousal)**

| Model Info | | |
| --- | --- | --- |
| **Info** |  |  |
| Model Type | Mixed Model | Linear Mixed model for continuous y |
| Model | lmer | `IES-R (IpA)` ~ 1 + Time + Treatment + Sex + Time:Treatment + Time:Sex + Treatment:Sex + Time:Treatment:Sex + ( 1 \| Subjects ) |
| Distribution | Gaussian | Normal distribution of residuals |
| Direction | y | Dependend variable scores |
| Optimizer | bobyqa |  |
| DF method | Satterthwaite |  |
| Sample size | 100 |  |
| Converged | yes |  |
| Y transform | none |  |
| C.I. method | Wald |  |

**Model Results**

| Model Fit | | | | |
| --- | --- | --- | --- | --- |
| **Type** | **R²** | **df** | **LRT X²** | **p** |
| Conditional | 0.687 | 8 | 76.428 | <.001 |
| Marginal | 0.472 | 7 | 76.428 | <.001 |

| Fixed Effects Omnibus Tests | | | | |
| --- | --- | --- | --- | --- |
|  | **F** | **df** | **df (res)** | **p** |
| **Time** | 139.90690 | 1 | 46.0 | <.001 |
| **Treatment** | 0.03967 | 1 | 46.0 | 0.843 |
| **Sex** | 0.10286 | 1 | 46.0 | 0.750 |
| **Time ✻ Treatment** | 0.42925 | 1 | 46.0 | 0.516 |
| **Time ✻ Sex** | 0.00183 | 1 | 46.0 | 0.966 |
| **Treatment ✻ Sex** | 2.58558 | 1 | 46.0 | 0.115 |
| **Time ✻ Treatment ✻ Sex** | 1.73596 | 1 | 46.0 | 0.194 |

| Parameter Estimates (Fixed coefficients) | | | | | | | | |
| --- | --- | --- | --- | --- | --- | --- | --- | --- |
|  | | | | **95% Confidence Intervals** | |  | | |
| **Names** | **Effect** | **Estimate** | **SE** | **Lower** | **Upper** | **df** | **t** | **p** |
| (Intercept) | (Intercept) | 1.00932 | 0.0857 | 0.839 | 1.180 | 46.0 | 11.7782 | <.001 |
| Time1 | pre - post | 1.31448 | 0.1111 | 1.094 | 1.535 | 46.0 | 11.8282 | <.001 |
| Treatment1 | 2 - 1 | 0.03413 | 0.1714 | -0.306 | 0.375 | 46.0 | 0.1992 | 0.843 |
| Sex1 | 1 - 0 | 0.05497 | 0.1714 | -0.286 | 0.395 | 46.0 | 0.3207 | 0.750 |
| Time1 ✻ Treatment1 | (pre - post) ✻ (2 - 1) | -0.14562 | 0.2223 | -0.587 | 0.296 | 46.0 | -0.6552 | 0.516 |
| Time1 ✻ Sex1 | (pre - post) ✻ (1 - 0) | -0.00951 | 0.2223 | -0.451 | 0.432 | 46.0 | -0.0428 | 0.966 |
| Treatment1 ✻ Sex1 | (2 - 1) ✻ (1 - 0) | 0.55118 | 0.3428 | -0.130 | 1.232 | 46.0 | 1.6080 | 0.115 |
| Time1 ✻ Treatment1 ✻ Sex1 | (pre - post) ✻ (2 - 1) ✻ (1 - 0) | 0.58568 | 0.4445 | -0.297 | 1.469 | 46.0 | 1.3176 | 0.194 |

| Random Components | | | | |
| --- | --- | --- | --- | --- |
| **Groups** | **Name** | **Variance** | **SD** | **ICC** |
| **Subjects** | (Intercept) | 0.208 | 0.456 | 0.408 |
| **Residual** |  | 0.302 | 0.550 |  |
| Nota. Number of Obs: 100 , Number of groups: Subjects 50 | | | | |

**Post Hoc Tests**

| Post Hoc comparison: Time ✻ Treatment | | | | | | | | | |
| --- | --- | --- | --- | --- | --- | --- | --- | --- | --- |
| **Comparison** | | | | |  | | | | |
| **Time** | **Treatment** | **vs** | **Time** | **Treatment** | **Difference** | **SE** | **t** | **df** | **p_bonferroni_** |
| post | 1 | - | post | 2 | -0.1069 | 0.204 | -0.524 | 78.9 | 1.000 |
| post | 1 | - | pre | 1 | -1.3873 | 0.156 | -8.915 | 46.0 | <.001 |
| post | 1 | - | pre | 2 | -1.3486 | 0.204 | -6.602 | 78.9 | <.001 |
| post | 2 | - | pre | 1 | -1.2803 | 0.204 | -6.268 | 78.9 | <.001 |
| post | 2 | - | pre | 2 | -1.2417 | 0.159 | -7.824 | 46.0 | <.001 |
| pre | 1 | - | pre | 2 | 0.0387 | 0.204 | 0.189 | 78.9 | 1.000 |
